# Transcription-dependent spreading of the Dal80 yeast GATA factor across the body of highly expressed genes

**DOI:** 10.1371/journal.pgen.1007999

**Published:** 2019-02-28

**Authors:** Aria Ronsmans, Maxime Wery, Ugo Szachnowski, Camille Gautier, Marc Descrimes, Evelyne Dubois, Antonin Morillon, Isabelle Georis

**Affiliations:** 1 Metabolism of Model Microorganisms, Labiris, Brussels, Belgium; 2 Laboratoire de Microbiologie, Université Libre de Bruxelles, Brussels, Belgium; 3 ncRNA, epigenetic and genome fluidity, Institut Curie, PSL Research University, CNRS UMR 3244, Université Pierre et Marie Curie, Paris, France; National Institute of Child Health and Human Development, NIH, UNITED STATES

## Abstract

GATA transcription factors are highly conserved among eukaryotes and play roles in transcription of genes implicated in cancer progression and hematopoiesis. However, although their consensus binding sites have been well defined *in vitro*, the *in vivo* selectivity for recognition by GATA factors remains poorly characterized. Using ChIP-Seq, we identified the Dal80 GATA factor targets in yeast. Our data reveal Dal80 binding to a large set of promoters, sometimes independently of GATA sites, correlating with nitrogen- and/or Dal80-sensitive gene expression. Strikingly, Dal80 was also detected across the body of promoter-bound genes, correlating with high expression. Mechanistic single-gene experiments showed that Dal80 spreading across gene bodies requires active transcription. Consistently, Dal80 co-immunoprecipitated with the initiating and post-initiation forms of RNA Polymerase II. Our work suggests that GATA factors could play dual, synergistic roles during transcription initiation and post-initiation steps, promoting efficient remodeling of the gene expression program in response to environmental changes.

## Author summary

GATA transcription factors are highly conserved among eukaryotes and play key roles in cancer progression and hematopoiesis. In budding yeast, four GATA transcription factors are involved in the response to the quality of nitrogen supply. Here, we have determined the whole genome binding profile of the Dal80 GATA factor, and revealed that it also associates with the body of promoter-bound genes. The observation that intragenic spreading correlates with high expression levels and exquisite Dal80 sensitivity suggests that GATA factors could play other, unexpected roles at post-initiation stages in eukaryotes.

## Introduction

In eukaryotes, gene transcription by RNA polymerase II (Pol II) is initiated by the binding of specific transcription factors to double-stranded DNA. The yeast transcription factors target regulatory regions called UAS or URS (for *U*pstream *A*ctivating/*R*epressing *S*equences), generally directly adjacent to the core promoter. The generated regulatory signals converge at the core promoter where they permit the regulation of Pol II recruitment via the ‘TATA box-binding protein’ and associated general transcription factors [1,2]. The transcription factor binding sites are usually short sequences ranging from 8 to 20 bp [3]. They are most often similar but generally not identical, differing by some nucleotides from one another [3], making it sometimes difficult to predict whether a given UAS will function as such *in vivo*.

GATA factors constitute a family of transcription factors highly conserved among eukaryotes and characterized by the presence of one or two DNA binding domains which consists of four cysteines (fitting the consensus sequence CX_2_CX_17-18_CX_2_C) coordinating a zinc ion followed by a basic carboxy-terminal tail [4]. While vertebrate GATA factors possess two adjacent homologous zinc fingers, fungal ones contain only one single zinc finger, being most closely related to the C-terminal vertebrate zinc finger [5,6], which is the one responsible for determining the binding specificity of GATA-1, the founding member of the GATA factor family [7]. The specificity of GATA factor binding has been thoroughly characterized in yeast [8–10] and metazoans [11–18]. In addition, structure determinations of protein-DNA complexes, first for GATA-1 [4], then for its fungal orthologue AreA [19], allowed for the identification of the subtle determinants of DNA specificity for GATA factors. Notably, the conserved DNA binding domain of GATA factors was reported to bind to consensus sequences (corresponding to GATAA(G) or GATTAG for the yeast GATA factors described hereafter), as shown in various organisms using direct or indirect methods [4,19–22]. These consensus sequences are accordingly referred to as GATA motifs.

Since its discovery 40 years ago in chicken cells, the family of GATA factors was extended in human cells and represents master regulators of hematopoiesis and cancer [23]. However, although approximately 7 million GATA motifs can be found in the human genome, the GATA factors occupy only 0.1–1% of them. Conversely, other regions are occupied by GATA factors despite lacking the consensus motif [24,25]. Consistently, even if most GATA factors bind to core GATA sequences, peculiar specificities have been reported for the flanking bases as well as for the fourth base of the GATA core element [26–29]. These studies revealed an elevated flexibility in the recognition sites for vertebrate and fungal GATA factors, much greater than previously anticipated, making the search for GATA sites and their enrichment in GATA-regulated genes tedious and unproductive. In addition, GATA factors can swap among them for the same motif and switch from active or repressive transcriptional activity. All these observations developed the main paradigm shift of how GATA factors are recruited and reside on the chromatin [30,31].

In yeast, the family of GATA transcription factors contains over 10 members [32]. Four of them are implicated in the regulation of *N*itrogen *C*atabolite *R*epression (NCR)-sensitive genes, the expression of which is repressed in the presence of a preferred nitrogen source (glutamine, asparagine, ammonia) and derepressed when only poor nitrogen sources (e.g. proline, leucine, urea) are available [10]. The key GATA factors involved in NCR signaling are two activators (Gln3 and Gat1/Nil1) and two repressors (Gzf3/Nil2/Deh1 and Dal80/Uga43) [33–38]. In a perfect feedback loop, the expression of *DAL80* and *GAT1* is also NCR-sensitive, which implies cross- and autogenous regulations of the GATA factors in the NCR mechanisms [38–41]. Under nitrogen limitation, expression of *DAL80* is highly induced [35], and Dal80 enters the nucleus where it competes with the two GATA activators for the same binding sites [20,39,42]. Although initially described as being active under nitrogen abundance [37,38], the Gzf3 repressor also localizes to NCR-sensitive promoters in conditions of activation [40].

The sequence conservation among the four yeast NCR GATA factors is remarkable and the residues involved in contacts with the DNA, thus specificity determination, are 100% conserved. In this respect, the binding sites of Dal80 on target DNA are likely to be recognized also by Gln3, Gat1 and Gzf3 [28]. *In vitro*, the Gln3 and Gat1 activators bind to single GATA sequences, presumably as monomers [43], like their orthologous vertebrate counterparts, while Dal80 was found to bind to two GATA sequences, 15–35 bp apart, in a preferred tail-to-tail orientation or to a lower extent in a head-to-tail configuration [9,20,39,44]. *In vivo*, GATA factor binding site recognition also appears to require repeated GATA motifs within promoters, as shown for the NCR-sensitive *DAL5* promoter [45–47]. This led to the actual fuzzy definition of *UAS*_*NTR*_, consisting in two GATA sites located close to one another to present a binding platform for GATA factors [45–47]. Finally, in some cases, the existence of auxiliary promoter sequences was shown to compensate single GATA site, allowing for transcriptional activation [48], although this was never as efficient as additional GATA sites [49]. The antagonistic role of Dal80 also requires multiple GATA sites [39,42], and inactivation of one of the four GATA sites of the *UGA4* promoter results in the loss of the Dal80-repressive activity while affecting moderately Gln3- and Gat1- activation capacity [20].

In summary, although NCR-sensitive genes are recognized to contain at least one GATA site, and often more, a precise definition of the minimal element required for binding and transcriptional regulation is still lacking.

In yeast, genome-wide ChIP analyses have allowed gaining insights into the GATA factor gene network through the identification of direct targets [50–53]. However, these studies were not performed in activating conditions, when all GATA factors are expressed, localized in the nucleus and active, so that the current list of GATA factor targets are likely to be underestimated. On another hand, bioinformatic analyses have shown that, since GATA sequences are short, they can be found almost everywhere throughout the genome. Therefore, based on the sole criteria of the presence of repeated GATA sequences in yeast promoters, a third of the yeast genes could hypothetically be NCR regulator targets [54]. However, such GATA motif repetitions have been found in the promoter of 91 genes, inducible by GATA activators in absence of a good nitrogen source, supposed to be directly targeted by the GATA activators [55]. Nevertheless, the functionality of these hypothetical UAS still needs to be directly demonstrated *in vivo* [1].

Here, we provide the first genome-wide identification of Dal80 targets in yeast, in physiological conditions where Dal80 is fully expressed and active. Using a ChIP-Seq approach combined to a bioinformatic peak-calling procedure, we defined the exhaustive set of Dal80-bound promoters, which turned out to be much larger than anticipated. Our data indicate that at some promoters, Dal80 recruitment occurs independently of GATA sites. Strikingly, Dal80 was also detected across the body of a subset of genes bound at the promoter, globally correlating with high and Dal80-sensitive expression. Mechanistic single-gene experiments confirmed the Dal80 binding profiles, further indicating that Dal80 spreading across gene bodies requires active transcription. Finally, co-immunoprecipitation experiments revealed that Dal80 physically interacts with active form of Pol II.

## Results

### Genome-wide identification of Dal80-bound promoters

**Fig 1:**
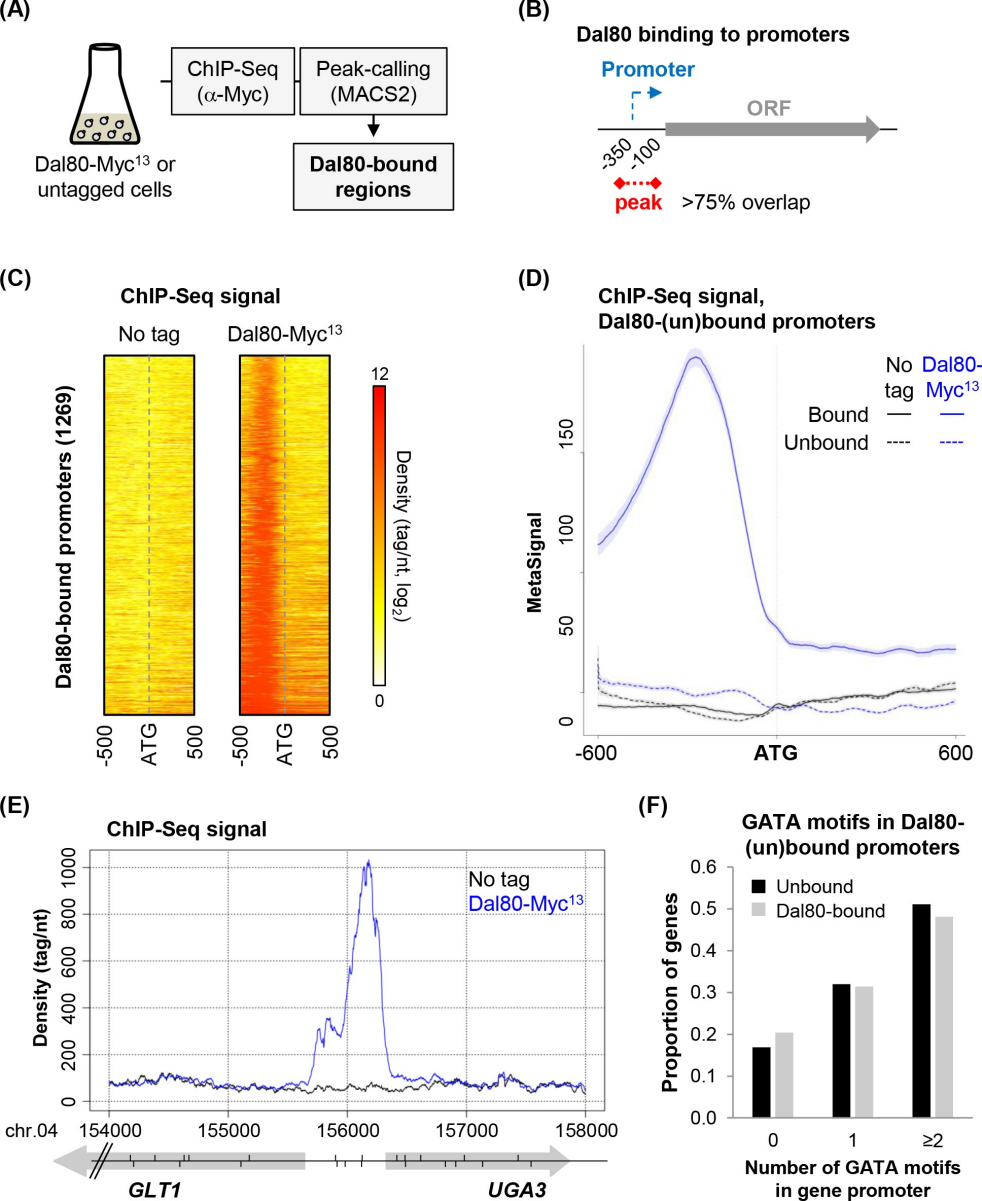
Genome-wide identification of Dal80-bound promoters. (A) Overview of the ChIP-Seq analysis. Biological duplicates of FV078 (*DAL80-MYC*^*13*^) and 25T0b (no tag) cells were grown to mid-log phase in proline-containing medium, and then harvested. After chromatin extraction and sonication, Dal80-Myc^13^ was immunoprecipitated using α-Myc antibody. Co-precipitated DNA fragments were purified and used to construct ChIP-Seq libraries. After sequencing of the libraries, signals were computed using uniquely mapped reads. Dal80-bound regions were identified using a peak-calling procedure using MACS2. (B) Identification of Dal80-bound promoters. After peak-calling, Dal80-bound promoters were identified on the basis of a >75% overlap of the -100 to -350 regions (relative to the downstream ORF start site) by the peak (represented as a red dashed line). (C) Heatmap view of the ChIP-Seq signal in the ATG +/- 500 bp region for the 1269 genes identified as bound by Dal80 at the promoter, in untagged and *DAL80-MYC*^*13*^ cells. (D) Metagene view of the ChIP-Seq signal along the ATG +/- 600 bp region for the 1269 genes identified as bound by Dal80 at the promoter (solid lines) and for the unbound genes (dashed lines), in untagged (black) and *DAL80-MYC*^*13*^ cells (blue). For each group of genes, normalized coverage (tag/nt) for each gene was piled up, and the average signal was plotted. The shading surrounding each line denotes the 95% confidence interval. (E) Snapshot of ChIP-Seq signals at the divergent *GLT1*/*UGA3* promoter region. Densities (tag/nt) are shown for the untagged (black line) and *DAL80-MYC*^*13*^ (blue line) strains. Genes are represented as grey arrows. The position (and orientation) of each GATA site is represented by vertical segments above (sense GATA sites) or below (antisense GATA sites) the *locus* line. The snapshot was produced using the VING software [94]. (F) Number of GATA (GATAA, GATAAG or GATTAG) sites in the promoter of Dal80-unbound and promoter-bound genes. The analysis was performed using RSAT [95], across the -500 to -1 region (relative to the ATG codon of the downstream ORF).

In order to determine the genome-wide occupancy of a GATA factor in yeast, our rationale was to choose Dal80 as it is known to be highly expressed in derepressing conditions and forms chromosome *foci* when tagged by GFP [56]. We grew yeast cells in proline-containing medium and performed a ChIP-Seq analysis using a Dal80-Myc^13^-tagged strain and the isogenic untagged strain, as a control (Fig 1A), after ensuring that the Myc^13^-tagged form of Dal80 was functional (S1A Fig). Dal80-bound regions were then identified using a peak-calling algorithm (see Material & Methods). A promoter was defined as bound by Dal80 on the basis of a >75% overlap of the -100 to -350 region (relative to the downstream ORF start site) by a peak (Fig 1B). We chose to use as the reference coordinate the translation initiation codon rather than the transcription start site (TSS) since the latter has not been accurately defined for all genes. Then, our arbitrary definition of the promoter as the -350 to -100 region relative to the ATG codon was based on the distribution of the TSS-ATG distance for genes with an annotated TSS (median and average distance = 58 and 107 bp, respectively; see S1B Fig).

Strikingly, Dal80 was found to bind to 1269 gene promoters (Fig 1C and 1D and S1 Table). This number, corresponding to 22% of all protein-coding gene promoters, is much higher than anticipated given the roughly hundred target genes generally cited for the GATA transcriptional activators Gat1 and Gln3 [55,57], presumably sharing binding sites with Dal80. However, we noted that some peaks (221) overlapped several promoters (471), mainly of divergent genes (442), as shown in Fig 1E for an illustrative example. Despite it is possible that in such cases, only one of the two divergent promoters is targeted by Dal80, the number of *in vivo* Dal80 target sites we identified here has been extensively extended from what was acknowledged so far.

Among the genes showing Dal80 binding at their promoter, we noticed a significant enrichment for cytoplasmic translation genes, as well as genes involved in small molecule biosyntheses, including amino acids (S2 Table). Before our work, very few studies have investigated the transcriptional targets of Dal80 *in vivo* in conditions of nitrogen deprivation. One of them, based on mini-arrays [58], identified 19 Dal80-regulated genes, all of which have been isolated in our ChIP-Seq analysis (highlighted in orange in column B of S3 Table). As expected given the similarity between binding sites of Dal80 and the other nitrogen-regulated GATA factors, other genes related to previous nitrogen regulation screens [55,57–64] are also significantly enriched within our list: 103 of the 205 previously identified nitrogen-regulated genes have been identified in our ChIP-Seq analysis using Dal80 as the bait, which is much more than expected by chance (*P*<0.001, Chi-square test; S3 Table, column B).

Surprisingly, analysis of GATA site occurrence over Dal80-bound and unbound promoters revealed no difference between the two classes, 48.2% and 51.3% of Dal80-bound and unbound promoters containing at least two GATA sites, respectively (Fig 1F). Likewise, we observed no major difference between the Dal80-bound and unbound promoters in respect of the GATA sites spacing (S1C Fig) and orientation (S1D Fig) preferences defined *in vitro* for Dal80 binding [9]. Intriguingly, 20% of Dal80-bound promoters do not contain any GATA site (Fig 1F), indicating that Dal80 recruitment can also occur independently of the presence of consensus GATA sites (see S1B Fig for visualization of Dal80 recruitment to a GATA-less promoter).

In summary, our ChIP-Seq analysis revealed that Dal80 binds to a set of promoters larger than previously expected, targeting biosynthetic functions and protein synthesis in addition to nitrogen catabolite repression.

### Dal80 recruitment to promoters correlates with nitrogen- and Dal80-sensivitiy

We asked whether Dal80-binding to promoters could be associated to regulation of gene expression by the nitrogen source and/or Dal80. We therefore performed RNA-seq in wild-type cells grown in glutamine- and proline-containing medium, and in *dal80Δ* cells grown in proline-containing medium.

**Fig 2:**
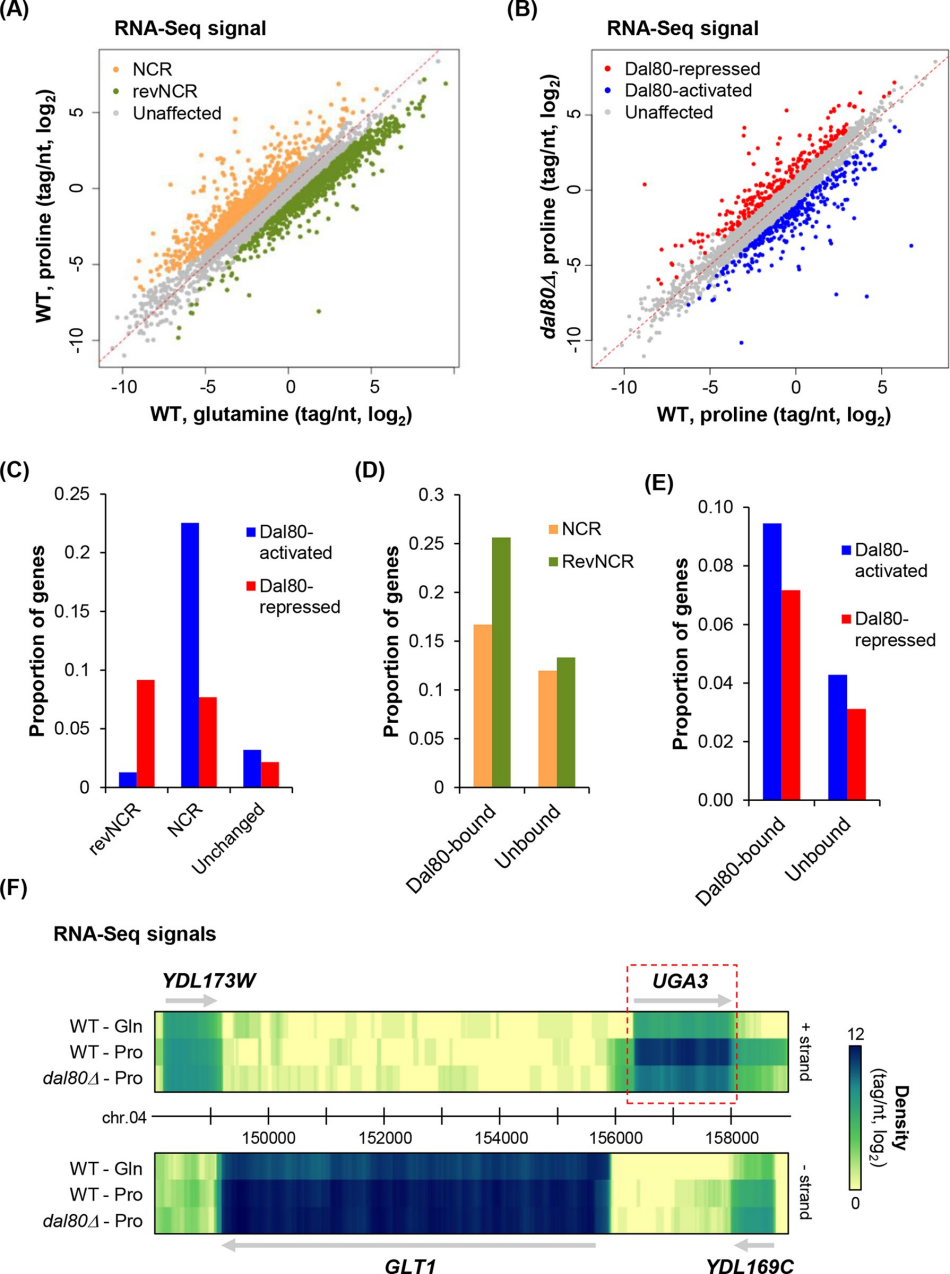
Dal80 recruitment to promoters correlates with nitrogen- and Dal80-sensitive gene expression. (A) Scatter plot of densities (tag/nt, log_2_ scale) for genes in wild-type (WT) cells grown in proline- or glutamine-containing medium. For each condition, total RNA was extracted from exponentially growing biological replicates of 25T0b (WT). After rRNA depletion, strand-specific RNA-Seq libraries were constructed and then sequenced. Tag densities were computed using uniquely mapped reads. NCR- and revNCR-sensitive genes were identified on the basis of a proline/glutamine ratio ≥2 or ≤0.5, respectively, with a *P*-value ≤0.01 upon differential expression analysis using DESeq [93]. Unaffected (4116), NCR-sensitive (754) and revNCR-sensitive (928) genes are shown as grey, orange and green dots, respectively. (B) Scatter plot of densities (tag/nt, log_2_ scale) for genes in 25T0b (WT) and FV080 (*dal80Δ*) cells grown in proline-containing medium. RNA extraction and construction of RNA-Seq libraries were as described above. Dal80-regulated genes were identified using a mutant/WT ratio ≥2 (Dal80-repressed) or ≤0.5 (Dal80-activated), with a *P*-value ≤0.01 upon differential expression analysis using DESeq [93]. Unaffected (n = 5252), Dal80-repressed (n = 232) and Dal80-activated (n = 314) genes are shown as grey, red and blue dots, respectively. (C) Proportion of Dal80-activated (blue bars) and Dal80-repressed (red bars) genes among revNCR-sensitive, NCR-sensitive and unchanged (*ie* neither revNCR nor NCR) genes. The numbers of genes among each group are presented in S2B Fig. *P* < 0.00001 upon Chi-square test of independence. (D) Proportion of NCR-sensitive (orange bars) and revNCR-sensitive (green bars) among Dal80-bound and unbound genes. See also S2C Fig for the numbers of genes among each group. *P* < 0.00001 upon Chi-square test of independence. (E) Proportion of Dal80-activated (blue bars) and Dal80-repressed (red bars) genes among Dal80-bound and unbound genes. See also S2D Fig for the numbers of genes among each group. *P* < 0.00001 upon Chi-square test of independence. (F) Snapshot of RNA-Seq signals for the NCR-sensitive, Dal80-activated gene *UGA3*. RNA-Seq signals are visualized as a heatmap. The upper and lower panels show the signals for the + and—strands, respectively. The color turns from yellow to dark blue as the signal increases (scale on the right). The *UGA3* mRNA is highlighted using the red box. The neighboring genes (*YDL173W*, *GLT1* and *YDL169C*) are also indicated. The snapshot was produced using the VING software [94].

Firstly, we identified 1682 (30%) genes differentially expressed (fold-change ≥2 or ≤0.5, *P* ≤0.01) in wild-type cells according to the nitrogen source provided (Fig 2A), including 754 genes upregulated (NCR-sensitive) and 928 downregulated (revNCR-sensitive) in proline-containing medium (see lists in S4 Table). Consistent with previous reports, *DAL80* was found in our set of NCR-sensitive genes (S4 Table), showing very low expression in glutamine-containing medium and strong derepression in proline (S2A Fig). More globally, 97 of the 205 genes previously identified as NCR-sensitive were also found in our list (*P*<0.0001, Chi-square test; S4 Table).

In parallel, we identified 546 genes showing significantly altered expression (fold-change ≥2 or ≤0.5, *P* ≤0.01) in proline-grown *dal80Δ* cells compared to wild type (Fig 2B; S5 Table). In agreement with the previously described repressive activity of Dal80 [35], 232 genes are indeed negatively regulated by Dal80 (up in *dal80Δ*; red dots in Fig 2B). Unexpectedly, 314 genes are positively regulated by Dal80 (down in *dal80Δ*; blue dots in Fig 2B). This is the first *in vivo* global indication suggesting a positive function for Dal80 in gene expression. The Dal80-repressed group was enriched for genes involved in small molecule catabolic processes (S6 Table), while the Dal80-activated genes were mostly involved in amino acid biosynthesis (S7 Table). Again, we noticed an overlap between Dal80-regulated genes and nitrogen regulated genes that were identified in other screens: 86 of the 205 previously identified nitrogen-regulated genes have been identified as Dal80-regulated, which is much more than expected by chance (*P*<0.0001, Chi-square test; column D of S3 Table).

Globally, we observed a significant correlation between Dal80-sensivity and regulation by the nitrogen source (*P*<0.00001, Chi-square test; Fig 2C; see also S2B Fig). Indeed, there are more NCR-sensitive Dal80-activated and Dal80–repressed genes than expected in case of independence (Fig 2C; see also S2B Fig). Similarly, the number of revNCR-sensitive Dal80-repressed genes is also significantly higher than expected by chance (Fig 2C; see also S2B Fig). In contrast, the number of revNCR-sensitive Dal80-activated genes is significantly lower than expected by chance (Fig 2C; see also S2B Fig), indicating a negative correlation in this case. This observation is consistent with the *DAL80* gene itself being NCR-sensitive, so that the Dal80-activated genes can only be activated when *DAL80* is expressed.

More importantly, Dal80 recruitment to promoters significantly correlated with nitrogen- and Dal80-sensitivity. In fact, nitrogen-regulated expression and Dal80-binding are not independent, as NCR-sensitive (212) and especially revNCR-sensitive (325) genes are significantly enriched in Dal80-bound genes (*P*<0.00001, Chi-square test; Fig 2D; see also S2C Fig). We also observed a significant correlation between Dal80-sensitive gene expression and Dal80 recruitment at the promoter: 211/546 of Dal80-regulated genes were bound by Dal80, including 120/314 Dal80-activated and 91 Dal80-repressed genes, which again is much more than expected by chance (*P*<0.00001, Chi-square test; Fig 2E; see also S2D Fig). Fig 2F shows an illustrative example of an NCR-sensitive, Dal80-activated gene (*UGA3*), the promoter of which is bound by Dal80 (Fig 1E). S3A Fig shows the RNA-Seq signals for another NCR-sensitive, Dal80-repressed and Dal80-bound gene (*MEP2*), correlating with Pol II occupancy levels (S3B Fig).

In summary, there is a significant correlation between Dal80 recruitment to the promoter of genes and a regulation by the nitrogen source and/or Dal80 at the RNA level, indicating that Dal80 recruitment to promoters is physiologically relevant. More specifically, we identified a subset of 211 Dal80-bound genes that are regulated by Dal80 (S3 Table), and that are therefore a robust class of direct Dal80 targets.

### Dal80 occupancy across the intragenic region of a subset of genes

The metagene analysis described above revealed that the genes bound by Dal80 at the promoter also display a signal along the gene body, although this intragenic signal remains globally lower than in the promoter-proximal region (Fig 1D). This observation prompted us to investigate the possibility that Dal80 also occupies the gene body, at least for a subset of genes.

**Fig 3:**
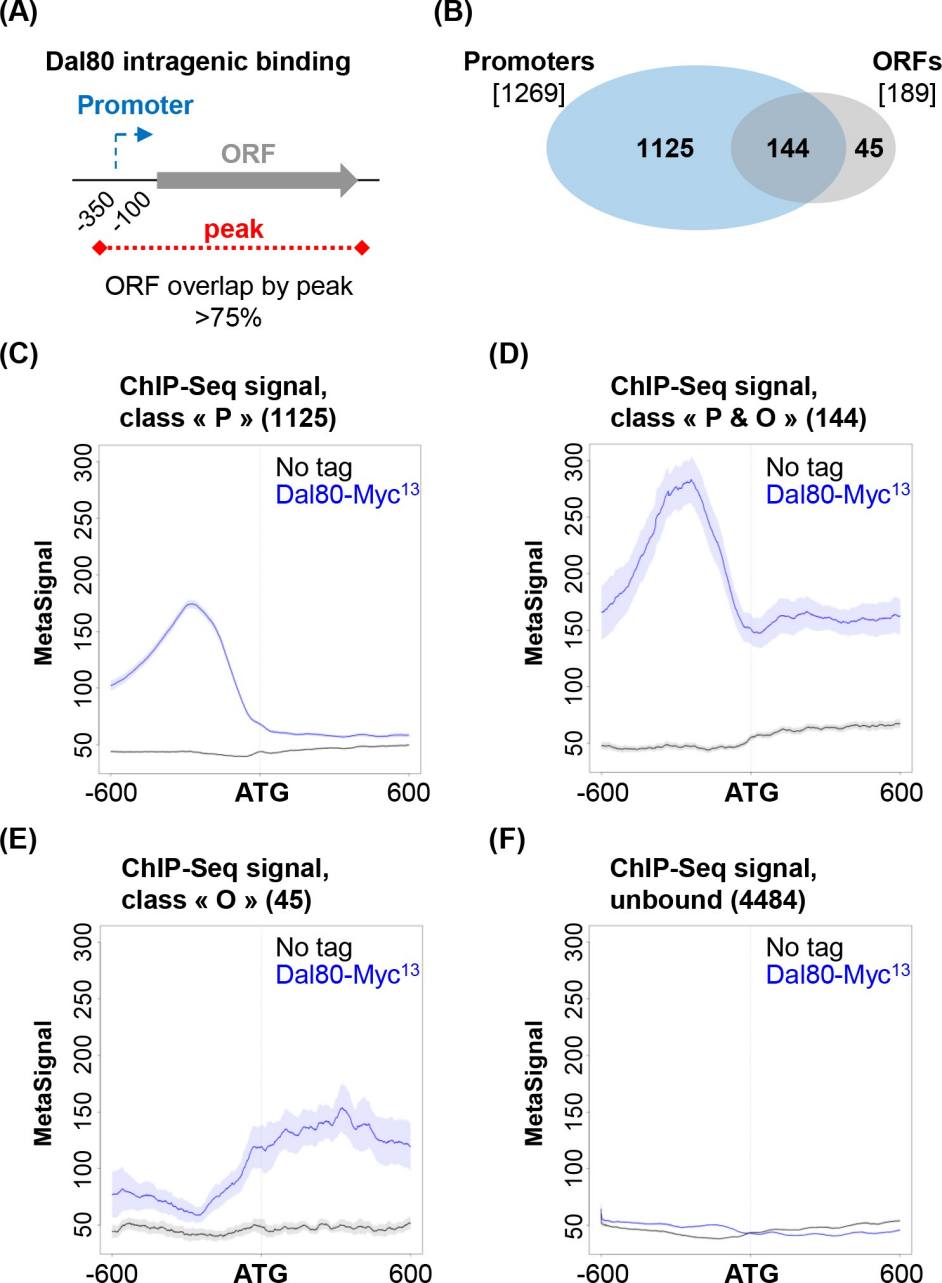
Dal80 spreading across the coding region of a subset of genes. (A) Identification of genes showing Dal80 intragenic binding. Dal80-bound ORFs were identified on the basis of a >75% overlap of the ORF by a Dal80-Myc^13^ peak (represented as a red dashed line). (B) Venn diagram showing the number of genes that are bound by Dal80 at the promoter and across the coding region (ORF). (C) Metagene view of the ChIP-Seq signal along the ATG +/- 600 bp region for the 1125 genes identified as bound by Dal80 at the promoter only (“P” class), in untagged (black) and *DAL80-MYC*^*13*^ cells (blue). MetaSignal computation was as described in Fig 1D. The shading surrounding each line denotes the 95% confidence interval. (D) Same as above for the 144 genes bound by Dal80 at the promoter and across the ORF (“P&O” class). (E) Same as above for the 45 genes bound by Dal80 across the ORF only (“O” class). (F) Same as above for the 4484 genes that are not bound by Dal80.

We identified 189 genes showing Dal80 intragenic occupancy, according to a >75% overlap of the ORF by a Dal80-Myc^13^ peak (Fig 3A and 3B). Among them, 144 (76%) were also bound at the promoter (Fig 3B). On the other hand, 45 genes showing Dal80 intragenic binding were not bound at the promoter (Fig 3B). Hence, we distinguished four classes of genes (S8 Table): (*i*) those bound by Dal80 at the promoter only (“P” class; Fig 3C; S8 Table, column C), (*ii*) those showing both promoter and intragenic binding (“P&O” class; Fig 3D; S8 Table, column E), (*iii)* those bound across the ORF only (“O” class; Fig 3E; S8 Table, column D), (*iv*) the unbound genes (Fig 3F). Interestingly, we noted that the global Dal80-Myc^13^ signal at the promoter was higher for the “P&O” class in comparison to the “P” class (Fig 3C and 3D).

Most of the genes of the “O” class are not Dal80-sensitive (40/45; S8 Table, column J). Furthermore, a substantial fraction of them correspond to small dubious ORFs, close to or even overlapping an adjacent Dal80-bound gene promoter. In these cases, the limited resolution of the ChIP-Seq technique, combined to the small size of these genes, might have allowed them to pass the filters we used to identify Dal80 intragenic binding. Overall, these observations suggest that the existence of the “O” class is likely to be physiologically irrelevant. Therefore, this class will not be further considered in our study.

In conclusion, we identified a subset of genes showing intragenic Dal80 occupancy, in most cases correlating with a strong Dal80 recruitment at the promoter.

### Dal80 occupancy across gene bodies correlates with high expression levels

**Fig 4:**
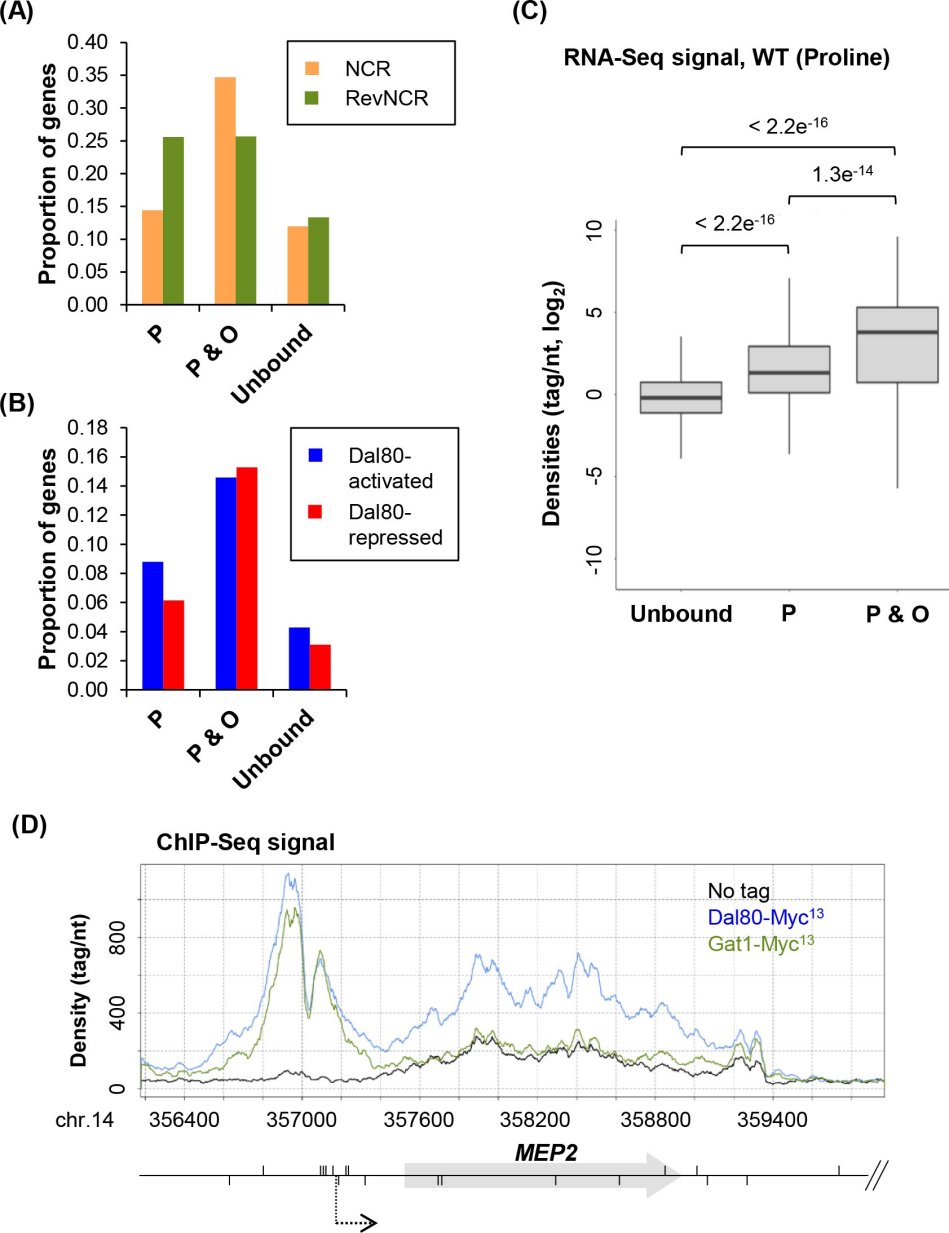
Dal80 spreading across gene bodies correlates with high expression levels. (A) Proportion of NCR-sensitive (orange bars) and revNCR-sensitive (green bars) among the “P”, “P&O” and unbound classes. See S4A Fig for the numbers of genes among each group. *P* < 0.00001 upon Chi-square test of independence. (B) Same as above for the Dal80-activated (blue bars) and Dal80-repressed (red bars) genes. See S4B Fig for the numbers of genes among each group. *P* < 0.00001 upon Chi-square test of independence. (C) Box-plot of densities (tag/nt, log_2_ scale) in wild-type cells grown in proline-containing medium, for genes of the unbound, “P” and “P&O” classes. *P*-values obtained upon Wilcoxon rank-sum test are indicated. (D) Snapshot of ChIP-Seq signals along the *MEP2 locus*. Densities (tag/nt) are shown for the untagged (25T0b; black line), *DAL80-MYC*^*13*^ (FV078; blue line) and *GAT1-MYC*^*13*^ (FV034; green line) strains, grown in proline-containing medium. Gene and GATA sites are represented as in Fig 1E. *MEP2* transcriptional start site (TSS) is indicated by a dashed arrow. The snapshot was produced using the VING software [94].

We asked whether Dal80 occupancy across gene bodies correlates with nitrogen-regulated gene expression and Dal80-sensitivity. We observed that nitrogen-regulated genes (NCR and revNCR; Fig 4A; see also S4A Fig) and Dal80-regulated genes (Dal80-activated and -repressed; Fig 4B; see also S4B Fig) were significantly more represented in the P&O class compared to the Dal80-unbound class.

Strikingly, we also observed that the genes of the P&O class are more expressed than the unbound genes (*P* < 2.2e^-16^, Wilcoxon rank-sum test; Fig 4C) but also than the P-bound genes (*P* = 1.3e^-14^, Wilcoxon rank-sum test; Fig 4C). However, it should be noted that a fraction of P-bound and unbound genes are expressed to higher levels than genes of the “P&O” class (S4C and S4D Fig), indicating that high expression does not always imply intragenic Dal80 occupancy.

Together with the observation that genes of the “P&O” class globally showed higher Dal80-Myc^13^ ChIP-Seq signal at the promoter than those of the “P” class (Fig 3C and 3D), our results indicate that Dal80 occupancy across gene bodies correlates with a stronger recruitment at the promoter and higher expression in proline-containing medium.

This raises the question of the specificity of the intragenic signal observed by ChIP-Seq. Indeed, for several proteins, unspecific ChIP signals have been detected across the body of a subset of highly expressed Pol II- and Pol III-dependent genes, referred to as ‘hyper-ChIPable’ *loci* [65–67]. We asked whether genes of our P&O class have been previously identified as ‘hyper-ChIPable’ (S9 Table, column G). This comparison indicated that 48/1125 of the P-bound genes and 27/144 of the P&O genes match with hyper-ChIPable *loci* (S4E and S4F Fig; see also S9 Table, columns H-I), suggesting that for a minority of cases, the intragenic Dal80 signal could be due to the ‘hyper-ChIPability’ of the *locus* and therefore be non-specific.

However, since these ‘hyper-ChIPable’ *loci* were defined under growth conditions that are different from those used in our study (growth in rich medium *vs* proline-containing synthetic medium), we aimed to get a more robust control for the specificity of Dal80 within gene bodies. Our rationale was to evaluate how similar and/or specific two close GATA factors could share/distinguish this “so called” artefactual hyper-ChIPability property. We performed a similar ChIP-Seq analysis using another GATA factor, the Gat1 activator [68], using the same conditions and following the same experimental procedure as described above (Figs 1A, 1B & 3A). Interestingly, 83.2% (936/1125) of the promoters bound by Dal80 were also bound by Gat1 (S4G Fig; S9 Table, column E), reinforcing the accuracy of the extended list of novel GATA-bound genes in yeast. Strikingly, the proportion of common targets among the P&O class dramatically decreased, 55% (79/144) of the genes bound by Dal80 at the promoter and across the gene body also showing promoter and intragenic binding for Gat1 (S4H Fig; S9 Table, column F). Importantly however, 65/144 P&O for Dal80 do not display intragenic binding for Gat1 (S4H Fig; S9 Table, column F), although Gat1 is recruited to the promoter of 57 of them. Thus, we can define a subset of 57 genes showing a specific intragenic occupancy of Dal80, while both Dal80 and Gat1 are recruited to their promoters similarly. As an illustrative striking example, Fig 4D shows a snapshot of the ChIP-Seq signals across *MEP2*, a well-characterized NCR-sensitive gene, the promoter of which is bound by the two GATA factors, but only Dal80 is found within the gene body.

To summarize, Dal80 occupancy across the gene body correlates with high expression levels. In a substantial proportion of cases, intragenic occupancy was found to be specific for Dal80, as another GATA factor also recruited to the promoter in the same experimental conditions was not detected within the gene body.

### Dal80 binding across the body of a well-characterized NCR-sensitive gene

**Fig 5:**
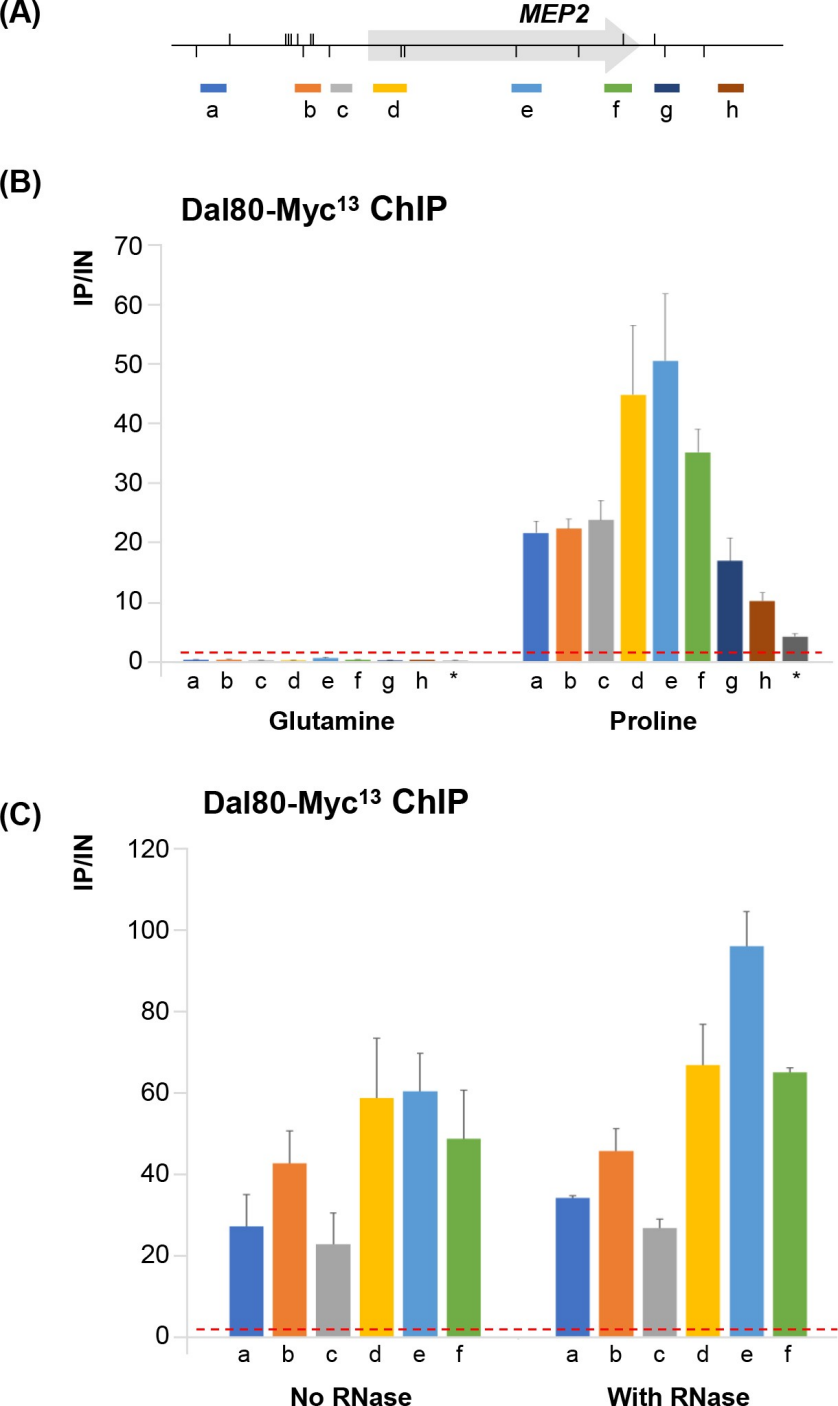
Dal80 binding across the body of a well-characterized NCR-sensitive gene. (A) Schematic representation of the *MEP2 locus* showing the position of the GATA sites (vertical segments above -sense GATA sites- or below -antisense GATA sites- the *locus* line) and qPCR probe positions (a, MEP2_P5-P6_; b, MEP2_P3-P4_; c, MEP2_P9-P10_; d, MEP2_O1-O2_; e, MEP2_O11-O12_; f, MEP2_O9-O10_; g, MEP2_D1-D2_; h, MEP2_D3-D4_). (B) Dal80-Myc^13^ occupancy across *MEP2*. Untagged (25T0b) and *DAL80-MYC*^*13*^ (FV078) cells were grown in the presence of glutamine or proline as unique nitrogen source. Anti-Myc ChIP was then performed as described in Materials and Methods. Each histogram represents the average (x10 000) of the value IP/IN (input or total chromatin) of at least three independent cultures on which two IPs were performed. The associated error bars correspond to the standard error. The red dashed line indicates the background signal obtained with the untagged strain. The last probe (indicated by the star) corresponds to the *DAL5* 2.5kb upstream region (DAL5U1-U2), used as an unbound control [68]. (C) Dal80-Myc^13^ occupancy across *MEP2* does not depend on RNA. Untagged (25T0b) or *DAL80-MYC*^*13*^ (FV078) cells were grown in proline-containing medium. ChIP analysis was conducted as above, with or without RNase treatment before the immunoprecipitation step. Each bar represents the average (x10 000) of the value IP/IN (input or total chromatin) of independent cultures of *DAL80-MYC*^*13*^ cells. Histograms represent the averages of at least 2 independent experiments and the associated error bars correspond to the standard error.

In order to validate our genome-wide observations and get additional mechanistic insights into the molecular bases of Dal80 occupancy across the body of highly expressed genes, we characterized the binding profile of Dal80 along the ammonium permease-coding gene *MEP2*, an NCR-sensitive gene of the “P&O” class (see Fig 4D). ChIP experiments followed by qPCR confirmed that Dal80 binds not only the promoter, but also across the coding region of *MEP2* in proline-grown cells (Fig 5A and 5B). No signal was observed in glutamine-grown cells (Fig 5B), indicating that Dal80 recruitment only occurs when it is expressed (S2A Fig).

To determine whether Dal80 intragenic occupancy is mediated by nascent RNA binding during transcription, we performed a similar ChIP experiment on the *MEP2* gene, treating the chromatin with RNase before the immunoprecipitation. Our results show no significant change of the Dal80-Myc^13^ signal across *MEP2* upon RNAse treatment of the chromatin extracts before the immunoprecipitation (Fig 5C), indicating that Dal80 occupancy across the gene body does not depend on RNA.

### Active transcription is required for Dal80 binding across gene body

**Fig 6:**
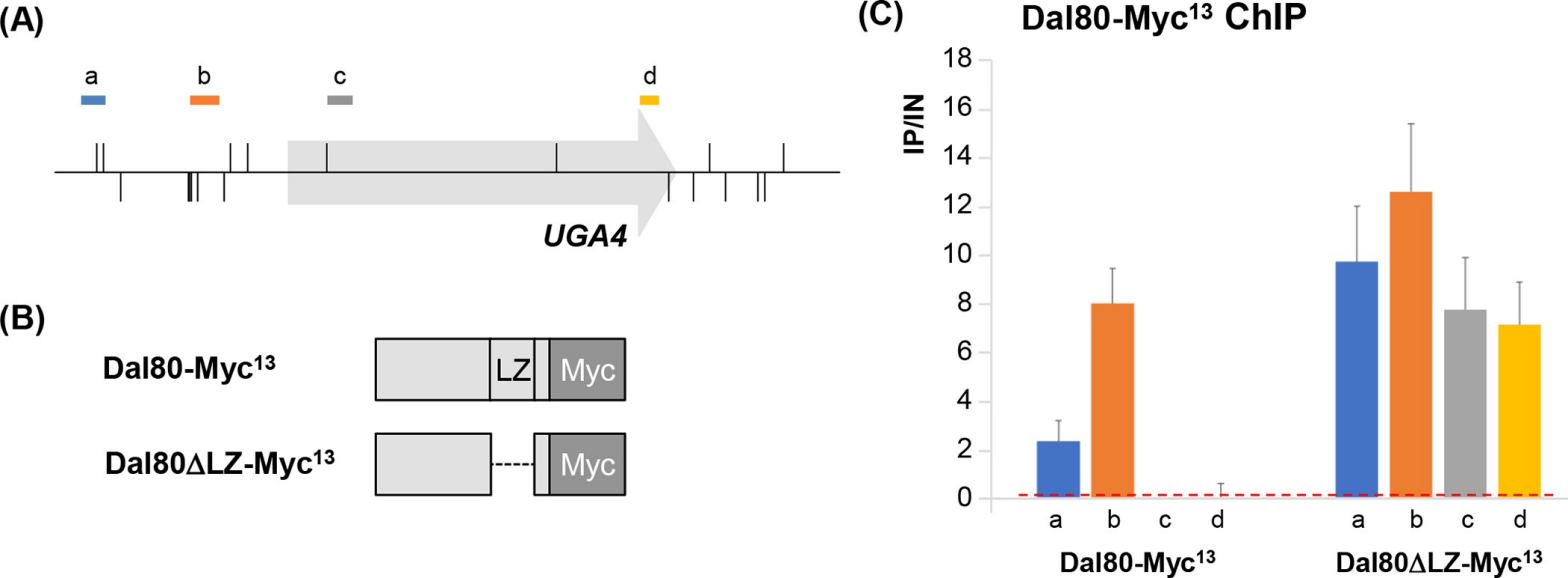
Dal80 occupancy across gene bodies requires active transcription and correlates with Pol II occupancy. (A) Schematic representation of the *UGA4 locus*, GATA sites (vertical segments above -sense GATA sites- or below -antisense GATA sites- the *locus* line) and qPCR probe positions (a, UGA4_P9-P10_; b, UGA4_P1-P2_; c, UGA4_O1-O2_; d, UGA4_O3-O4_). (B) Schematic representation of full-length and truncated (*Δ*LZ) versions of Dal80-Myc^13^. (C) *DAL80-MYC*^*13*^ (FV078) and *DAL80ΔLZ-MYC*^*13*^ (FV136) cells were grown in proline-containing medium. ChIP analysis was performed as described in Fig 5B with primers described in (A). Histograms represent the averages of at least 2 independent experiments and the associated error bars correspond to the standard error.

Since genes of the Dal80 “P&O” class are globally highly expressed, we asked whether active transcription is a prerequisite for Dal80 binding across the ORF. Our strategy was to select an NCR gene for which Dal80 is bound at the promoter when repressed and then monitor Dal80 occupancy once the gene is activated. Our RNA- and ChIP-Seq data allowed us to isolate the *UGA4 locus*, another well-characterized NCR-sensitive gene, bound by Dal80 at the promoter (Fig 6A; see snapshot in S5A Fig). *UGA4* expression is induced by GABA (γ-aminobutyric acid) and is strongly repressed by Dal80 in the absence of the inducer [69]. To derepress *UGA4* without inducer, a Dal80-specific deletion in the C-terminal leucine zipper domain was generated, impairing Dal80 repressive activity without affecting its binding capacity [34,44]. Indeed, in the Dal80*Δ*LZ-Myc^13^ strain (Fig 6B), the steady-state level of *UGA4* mRNA (S5B Fig) and Pol II occupancy (S5C Fig) both increased to derepressed levels in non-inducing conditions, like in a *dal80Δ* strain. Strikingly, in these conditions, full-length Dal80-Myc^13^ binding was restricted to the *UGA4* promoter (Fig 6A; see also S5A Fig), while Dal80*Δ*LZ-Myc^13^ binding was detected at the promoter and across the body of *UGA4* (Fig 6A). Interestingly, the leucine zipper of Dal80 and consequently, its dimerization, needed for *UGA4* repression, were not required for its localization across the *UGA4* gene body. Importantly, these results confirm that promoter binding is not sufficient to confer intragenic binding, but suggest that transcription activation is required.

Altogether, these observations prompted the important mechanistic question of how Dal80 can be localized to gene bodies upon transcription activation.

### Dal80 occupancy within gene bodies requires NCR promoter binding and correlates with Pol II occupancy

**Fig 7:**
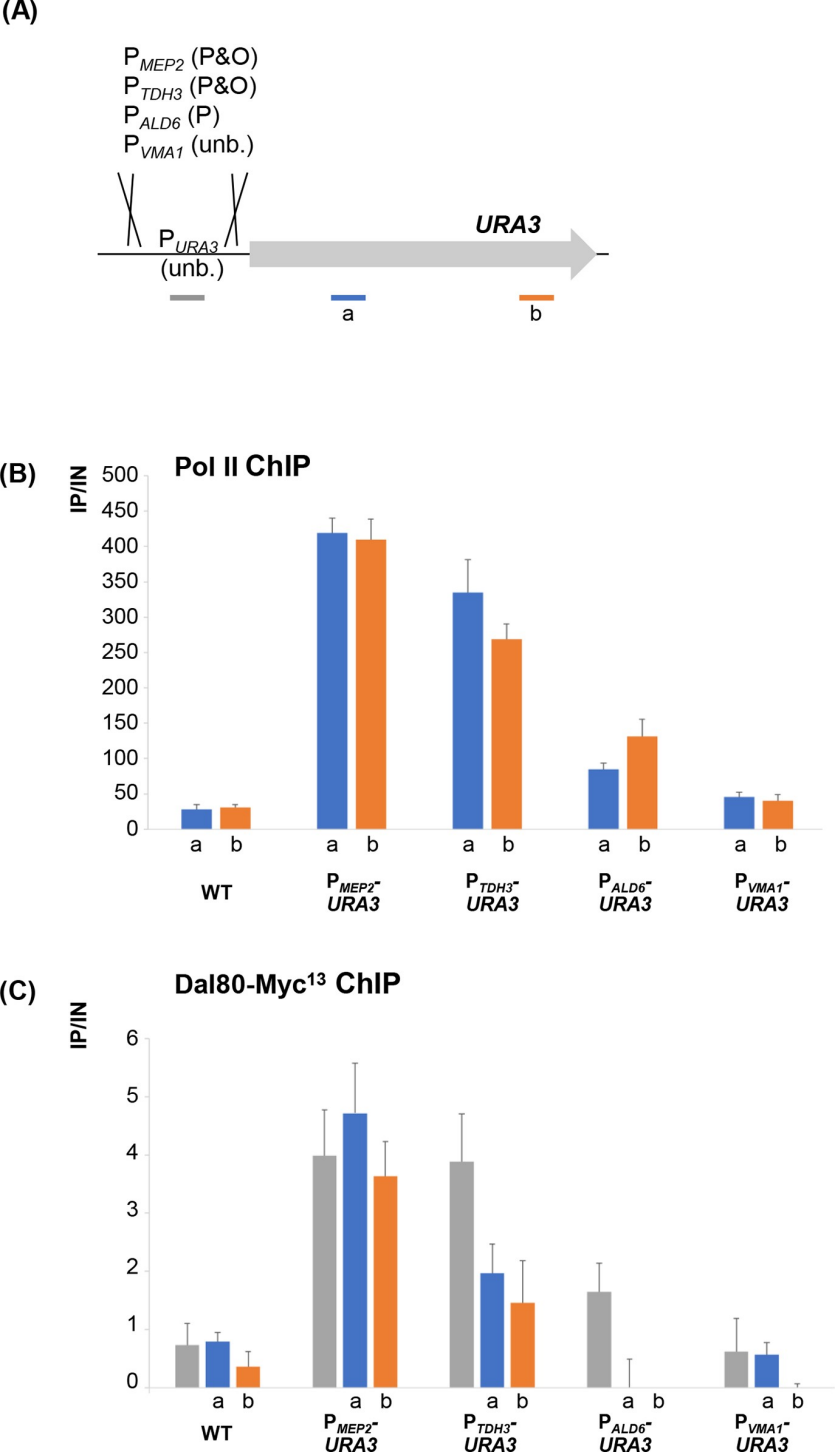
Dal80 occupancy within gene bodies requires NCR promoter binding and correlates with Pol II occupancy. (A) Schematic representation of the promoter fusions analyzed. Wild type *URA3 locus* was compared to *loci* in which the *URA3* open reading frame was inserted downstream of the *MEP2*, *TDH3*, *ALD6* or *VMA1* promoter regions. Promoter- and ORF-bound (P&O), promoter only-bound (P) and unbound (unb.) genes are indicated. Primers specific for each promoter (URA3_P1-P2_, MEP2_P9-P10_, TDH3_P1-P2_, VMA1_P1-P2_ and ALD6_P1-P2_) were used (grey segment) in the corresponding strains, as well as URA3_O1-O2_ (a) and URA3_O3-O4_ (b). (B) Occupancy of the *URA3* ORF by Pol II. WT (FV078), P_*MEP2*_-*URA3* (FV808), P_*TDH3*_-*URA3* (FV1105), P_*ALD6*_-*URA3* (FV1107) and P_*VMA1*_-*URA3* (FV1106) *DAL80-MYC*^*13*^ cells were grown in proline-containing medium. Anti-Pol II (CTD4H8) ChIP-qPCR analysis was performed as described in Fig 5B, using primers described in (A). (C) Occupancy of the *URA3* ORF and the upstream promoter by Dal80-Myc^13^. WT (FV078), P_*MEP2*_-*URA3* (FV808), P_*TDH3*_-*URA3* (FV1105), P_*ALD6*_-*URA3* (FV1107) and P_*VMA1*_-*URA3* (FV1106) *DAL80-MYC*^*13*^ cells were grown in proline-containing medium. ChIP-qPCR analysis was performed as described in Fig 5B, using primers described in (A).

In order to test if the presence of an NCR-sensitive promoter could confer intragenic Dal80 binding across the body of a non-NCR-sensitive gene, we placed the *URA3* ORF under the control of different promoters bound or not by Dal80: the *MEP2* and *TDH3* promoters as P&O representative, the *ALD6* promoter for the P class and the *VMA1* promoter, which is not bound by Dal80 (Fig 7A). When driven by P_*MEP2*_, the expression of *URA3* becomes NCR-sensitive and followed wild-type *MEP2* expression (S6 Fig), correlating with Pol II recruitment over the *URA3* ORF (Fig 7B). In these conditions, we observed Dal80-Myc^13^ binding at the promoter of *MEP2* and also across *URA3* (Fig 7C). Similarly for *P*_*TDH3*_*-URA3* construct, Dal80 also was relocalized within the *URA3* ORF, although to a lesser extent. Importantly, Dal80 binding was not detected across *URA3* when it was expressed from its native *locus*, under the control of its promoter (Fig 7C) or under the control of the Dal80-bound P_*ALD6*_ or unbound P_*VMA1*_ (Fig 7C), reinforcing the idea that those promoters fail to carry sufficient information for Dal80 to occupy the *URA3* ORF. Among the obvious characteristics, we noticed that Pol II occupancy is higher within those P&O *URA3* genes than the P only, suggesting that transcription strength might be a key determinant for Dal80 localization across the ORF. Interestingly, among the P&O fusions (*MEP2* and *TDH3*), we noted a difference in Dal80 binding levels to the adjacent *URA3* ORF, while those of Pol II remain similar across the two coding regions, suggesting that Pol II level might not be the only factor that control Dal80 occupancy.

In conclusion, these results show that for the same *URA3* sequence, the Dal80 occupancy displays distinct features depending only on the promoter characteristics to be classified as P, P&O or unbound, reflecting transcriptional strength. We propose that Dal80 presence within the ORF could be attributed to a spreading mechanism, controlled by Pol II complex and Dal80-promoter recognition capacity. These results exclude strongly DNA motif(s) as a main determinant for Dal80 spreading into ORF but rather raise the question of the direct implication of Pol II itself.

### Pol II interacts with Dal80 and its integrity is necessary for Dal80-spreading across *MEP2*

**Fig 8:**
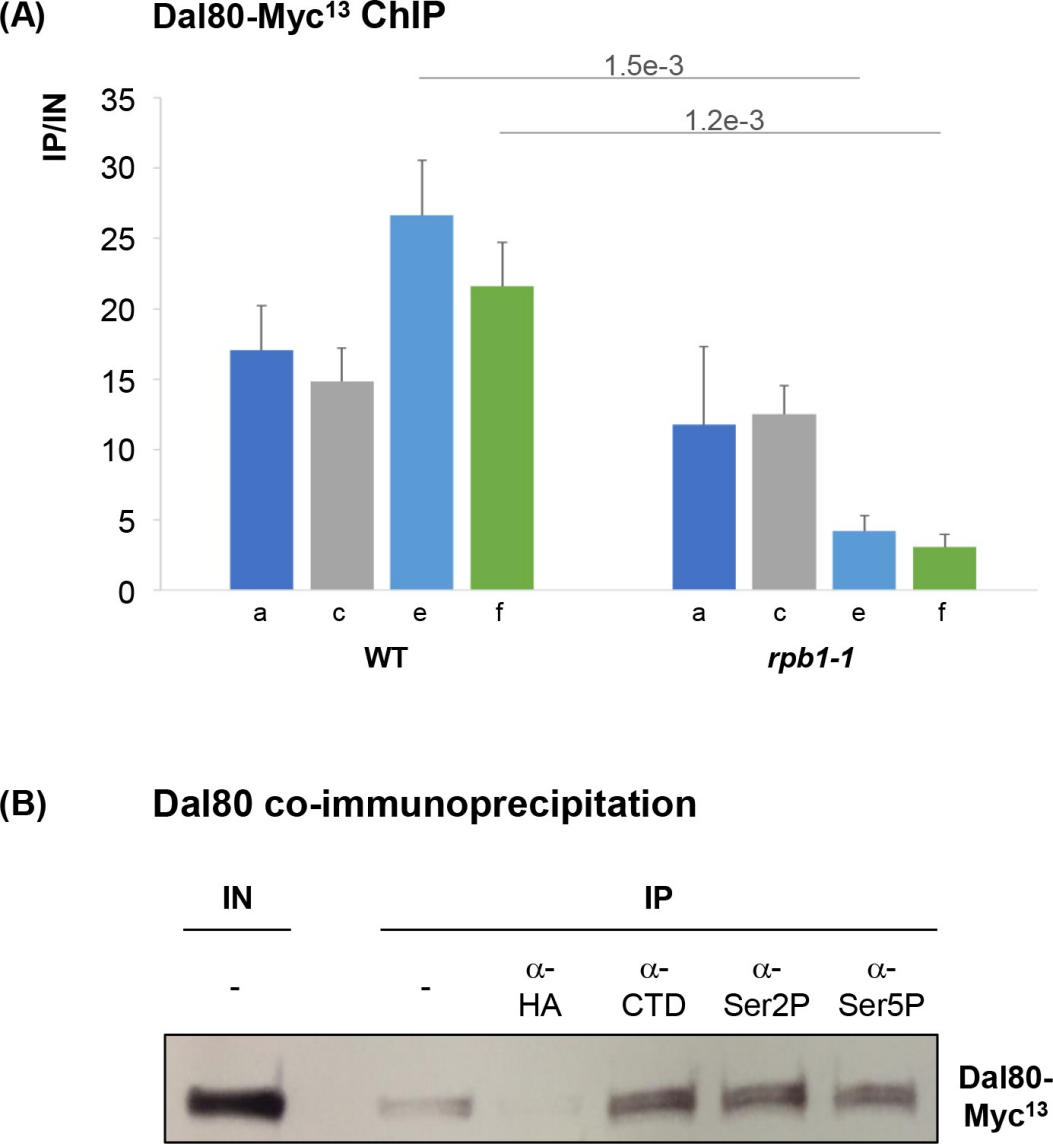
Dal80 binding across *MEP2* depends on active transcription. (A) Wild type (FV673) or *rpb1-1* (FV675) *DAL80-MYC*^*13*^ cells were grown to mid-log phase at 29°C in the proline-containing medium, and then shifted at 37°C for one hour. ChIP analysis was conducted as in Fig 5B, with primers described in Fig 5A. Histograms represent the averages of at least 2 independent experiments and the associated error bars correspond to the standard error. (B) Coimmunoprecipitation of Dal80-Myc^13^ with different phosphoforms of Pol II. Total proteins were extracted, immunoprecipitated with the indicated antibodies, and subjected to anti-Myc western blot analysis.

To test the hypothesis that the active Pol II complex could be responsible for Dal80 spreading beyond Dal80-bound promoters, we assessed the effect of rapid inactivation of Pol II using the thermosensitive *rpb1-1* strain [70,71]. We analyzed Dal80-Myc^13^ binding along *MEP2* in WT and *rpb1-1* cells. When *rpb1-1* cells were shifted at 37°C for 1h, *MEP2* mRNA and Pol II levels showed a 2-fold (S7A Fig) and >10-fold decrease (S7B Fig), respectively, reflecting the expected transcription shut-down when *rpb1-1* cells are shifted in non-permissive conditions. In the same conditions, we observed a significant >5-fold reduction of Dal80-Myc^13^ levels across the *MEP2* ORF, while the binding at the promoter was not affected (Fig 8A). This result reinforces the idea that Dal80 spreading across the body of NCR-sensitive genes is strongly correlated to an active Pol II.

To get insights into the mechanism by which Dal80 associates to actively transcribed gene bodies, we tested whether it physically interacts with the transcriptionally engaged form of Pol II (Fig 8B). Total protein extracts from Dal80-Myc^13^ cells were immunoprecipitated with antibodies directed against the Pol II CTD and its phospho-forms Ser2P and Ser5P, respectively characteristic of elongating and initiating Pol II forms. All three antibodies enabled effective immunoprecipitation, whereas no antibody and nonspecific antibody controls generated a lower or no signal at all. Thus, Dal80 would physically interact with phosphoforms of the Pol III, suggesting a strong association with Pol II engaged in active transcription from initiating to elongating polymerase.

Together, our data indicate that Dal80 spreading across the body of NCR-sensitive genes depends on active transcription and that Dal80 interacts with the transcriptionally active forms of Pol II, supporting a model where Dal80 spreading across the body of highly expressed, NCR-sensitive genes might be the result of Dal80-Pol II association at post-initiation transcription phases.

## Discussion

Eukaryotic GATA factors belong to an important family of DNA binding proteins involved in development and response to environmental changes in multicellular and unicellular organisms, respectively. In yeast, four GATA factors are involved in Nitrogen Catabolite Repression (NCR), controlling gene expression in response to nitrogen source availability. One of them, the Dal80 repressor, itself NCR-sensitive, acts to modulate the intensity of NCR responses.

Over the past decade, a number of studies have screened the genome aiming at gathering an inventory of genes regulated by the nitrogen source. Although >500 genes have been shown to be differentially expressed upon change of the nitrogen source [57,64], the list of NCR-sensitive genes was reduced to about 100, based on their sensitivity to GATA factors [55,57,60,63], suggesting that the number of Dal80 targets would be situated in that range. Here, using ChIP-Seq, we identified 1269 Dal80-bound promoters, which considerably extends the list of potential Dal80 targets. In fact, the number of Dal80-bound promoters could even have been greater. Indeed, the GATA consensus binding site is rather simple and short, so that in yeast, a total number of 10,000 putative binding sites can be found in all protein-coding gene promoters, 2930 promoters having at least two GATA sites, which is thought to be a prerequisite for *in vivo* binding and function of the GATA factors. The difference between the number of promoters with ≥2 GATA sites and the number of Dal80-bound promoters suggests the existence of a selectivity for Dal80 recruitment. This selectivity could rely on promoter architecture and/or chromatin structure, conditioning the requirement for auxiliary DNA binding factors that would stabilize Dal80 at some promoters. Moreover, although we observed a significant correlation between Dal80 binding and regulation, the expression of most of the Dal80-bound genes was not affected in a *dal80Δ* mutant strain. Again, Dal80-dependence for transcribing these genes, as well as their NCR sensitivity, could require the presence of yet unknown cofactors which are not produced or inactive under the tested growth conditions. In mammals, GATA factors also display an extraordinary complexity in the relationships between binding and expression regulation. Like Dal80, GATA-1 and GATA-2 only occupy a small subset of their abundant binding motif throughout the genome, and the presence of the conserved binding site is insufficient to cause GATA-dependent regulation in most instances [72]. GATA-1 binding kinetics, stoichiometry and heterogeneous complex formations, conditioned by composite promoter architecture, influence its transcriptional activity and hence diversify gene expression profiles [72].

Given the high conservation at the amino acid level between the DNA binding domains of the four yeast NCR GATA factors, it is likely that they all recognize identical sequences (GATAA, GATAAG or GATTAG). This consensus has been largely validated in the past using gene reporter experiments, mutational analyses and *in vitro* binding experiments on naked DNA. Nonetheless, of the 1269 bound promoters, 48% contained at least two GATA sites, a proportion that is not different from that observed among unbound promoters, and the amount of GATA sites per promoter was not different between the two groups either. In addition, Dal80 recruitment was found to occur independently of the presence of GATA sites in 20% of Dal80-bound promoters, as also previously observed in mammalian cells [24,73]. Future experiments will be required to decipher how Dal80 can be recruited to these GATA-less promoters. Among the different possibilities is a recruitment of Dal80 by degenerated GATA motifs. In this regard, we identified 5 degenerated GATA motifs within a 70 bp window corresponding to the peak of Dal80 binding signal at the promoter of the GATA-less, Dal80-sensitive gene *ALD6* (see S1E Fig). However, it also has to be noted that upon tolerance of only one mismatch within the GATA consensus, multiple degenerated motifs are detected in every yeast promoter.

Unexpectedly, although Dal80 has always been described as a repressor, we identified 314 genes that are positively regulated by Dal80 (their expression is significantly decreased upon Dal80 deletion; S5 Table). These genes are significantly enriched in amino acid biosynthetic processes, resembling the amino acid starvation response mediated by the Gcn4 transcriptional activator. Interestingly, the promoter of 122/314 Dal80-activated genes contain Gcn4-binding sites (S5 Table), and this group of 314 Dal80-activated genes is significantly enriched for genes regulated by the General Amino Acid Control (GAAC; YeastMine Gene List, Publication Enrichment, *P*<1.6e-13), through the Gcn4 activator. Interconnections between NCR and GAAC have already been demonstrated, mostly at the level of nitrogen catabolism control: 1-a large number of non-preferential nitrogen sources leads to increased transcription of GAAC targets [57]; and 2- Gcn4 contributes, with Gln3, to the expression of some but not all NCR-sensitive genes [74,75]. However, this is the first time that evidence are provided indicating a positive role for Dal80 at the level biosynthetic gene expression.

The most striking and unexpected finding of this work is the observation that Dal80 also occupied the body of a subset of genes. Dal80 binding at the promoter and spreading across the body of the 144 genes of the “P&O” class correlated with high expression levels and sensitivity to Dal80. It has been previously reported that at some *loci*, referred to as ‘hyper-ChIPable’, high expression levels might induce artefactual detection of DNA-binding factors across gene bodies [65]. However, in the context of this work, several observations argue for a specific association of Dal80 with gene bodies, at least for a subset of genes. Firstly, a considerable fraction of genes of the “P” class show similar or even higher expression levels than genes of the “P&O” class (S4C and S4D Fig), indicating that high expression does not always induce spreading of Dal80 across the gene body. Secondly, only 27 of the genes of our “P&O” class have been previously defined as ‘hyper-ChIPable’ (S9 Table, column I), even if the conclusion should be taken with caution as the two sets of experiments were performed upon very distinct physiological conditions. Thirdly, and more importantly, a similar ChIP-Seq analysis performed under the same experimental conditions using another GATA factor (the Gat1 activator) allowed us to define a subset of 57 genes that are specifically and only bound by Dal80 across their body, while both Dal80 and Gat1 are recruited to their promoter (see Fig 4D and S4H Fig). Thus, although we cannot exclude that in few cases, the signals for Dal80 across the intragenic region could still depend on the hyper-ChIPability of the *locus*, we propose that for the majority of “P&O” genes, the intragenic association of Dal80 is specific and biologically relevant. This is further supported by the observation that Dal80-sensitive (-activated and–repressed) genes are statistically more enriched within the “P&O” class, compared to the “P” class (Fig 4B). However, the causality relationship between Dal80 intragenic binding and high expression levels in derepressing conditions (proline) remains unclear to date.

**Fig 9:**
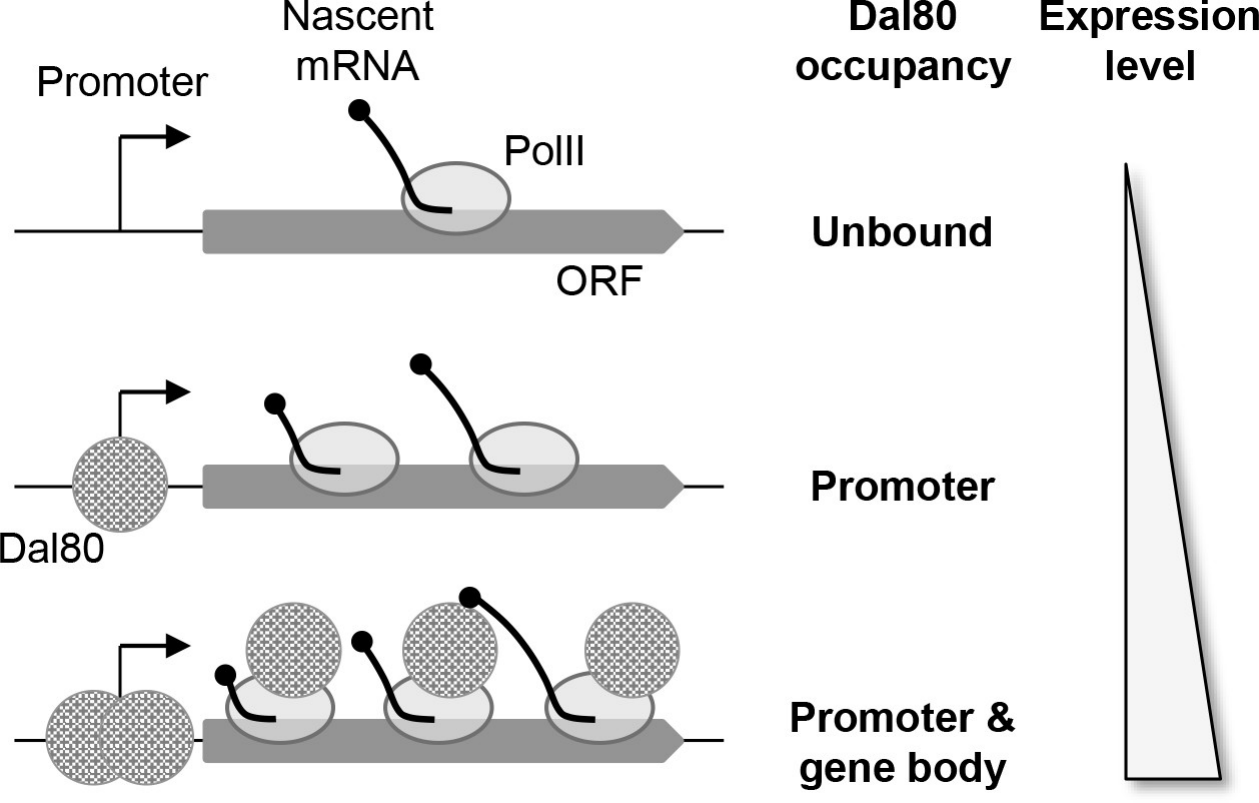
Model. See main text for details.

The observations we made at the genome-wide level were experimentally confirmed using ChIP experiments, at the level of single well-characterized NCR-sensitive genes. Promoter binding appears to be required but not sufficient. Indeed, the inactivation of Pol II-dependent transcription correlates with decreased intragenic binding (and *vice versa*), further indicating that Dal80 spreading across gene bodies depends on active transcription. Consistently, we detected a physical interaction between Dal80 and transcriptionally active forms of Pol II. Together, our data lead us to propose a model where Dal80 could travel from the promoter of highly expressed, NCR-sensitive genes through the gene body by accompanying the elongating Pol II complex (Fig 9). However, it is also possible that Dal80 spreading across gene bodies is determined, but yet temporally distinct, from the passage of the elongating Pol II. For instance, chromatin marks deposited upon Pol II passage could favor Dal80 intragenic binding afterwards. Additional investigations will be required to define which domain of Dal80 is responsible for the interaction with the transcription machinery, to determine whether there is any causal relationship between Dal80 intragenic binding and high expression levels, and to decipher the potential role of Dal80 during active transcription. In this respect, we propose that the leucine zipper domain is not involved.

Whereas the binding of elongation factors across gene bodies has been thoroughly documented [76], it has also been described for some specific transcription factors. For example, Gal4 was reported to bind to its consensus DNA target within the *ACC1* ORF, but the authors concluded that the observed transcriptional repression of the *ACC1* gene was most likely resulting from random *GAL4* binding “noise” over the genome, thus having no physiological explanation for this ORF-bound transcription factor [77]. Likewise, Gcn4 was detected across the *PHO8* ORF, with concomitant recruitment of the SAGA complex, but without any impact on gene expression [78]. More recently, binding of the Gcn4 transcription factor to its consensus site at some ORFs, when located in proximity of the transcriptional start site, was found to play a consistent role in controlling embedded cryptic promoters in yeast, thereby affecting Gcn4-dependent transcription of some genes [79].

A recent study has identified CTD phosphorylation of Pol II as a hub that optimizes transcriptome changes to adequately balance optimal growth and stress tolerance responses [80]. The addition of nitrogen to nitrogen-limited cells rapidly results in the transient overproduction of transcripts required for protein translation (stimulated growth) whereas accelerated mRNA degradation favours rapid clearing of the most abundant transcripts, like those involved in high affinity permease production, that are highly expressed NCR-sensitive genes, for example [64]. The involvement of the Nrd1-Nab3-Sen1 (NNS) and TRAMP complexes in these regulatory responses has been envisioned very recently [81,82]; deadenylation, decapping and exonuclease mutants display impaired *GAP1* mRNA clearance upon nitrogen upshift [83]. Thus, a possible role of Dal80 (and possibly of the other GATA factors) binding along highly expressed genes could be to transmit nutritional signals to elongation-related processes, like histone modification, chromatin remodelling [84,85], mRNA export/processing [86] or roadblock termination [87].

Interestingly, in human cells, GATA factors are also reported to occupy non-canonical sites within the genome, further reinforcing that they can be recruited to the chromatin independently of their motif [24,73]. In addition, 43% of the GATA1 peaks were collected among exon, introns and 3’UTR of coding genes in human erythroleukemia cells [73]. It is tempting to hypothesize that GATA factors could have a dual or synergistic role during transcription, i.e. recruiting/stabilizing the PIC complex as for any classical transcription factor in the promoter/enhancer regions and promoting competent transcription at a post initiation step interacting with the RNAPII.

## Materials and methods

### Experimental model and subject details

Experiments were conducted using *S*. *cerevisiae* strains of the FY genetic background. The strains used are listed in S10 Table. Dal80 and Gat1 were tagged with 13 copies of the *c-myc* epitope (Myc^13^) as described [88] using primers listed in S10 and S11 Tables. The *P*_*MEP2*_*-URA3* allele in strains FV806-808, and *P*_*TDH3*_*-URA3*, *P*_*VMA1*_*-URA3*, *P*_*ALD6*_*-URA3* alleles in strains FV1105-1107, respectively, were created by amplification of the *URA3* gene using the same strategy, with primers listed in S10 and S11 Tables.

Cultures were grown at 29°C to mid-log phase (A_660nm_ = 0.5) in YNB (without amino acids or ammonia) minimal medium containing the indicated nitrogen source at a 0.1% final concentration, glucose (3%) and the appropriate supplements (20 μg/ml uracil, histidine and tryptophan) to cover auxotrophic requirements.

### Chromatin immunoprecipitation

Cell extracts and chromatin immunoprecipitations were conducted as described [40] using primers listed in S11 Table. The cells (100 ml cultures grown to an absorbance (A660 nm = 0.6) corresponding to 6 × 10^6^ cells/ml) were treated with 1% formaldehyde for 30 min at 25°C and mixed by orbital shaking. Glycine was then added to a final concentration of 500 mM and incubation continued for 5 min. The cells were collected, washed once with cold 10 mM Tris-HCl, pH 8, washed once with cold FA-SDS buffer (50 mM HEPES-KOH, pH 7.5, 150 mM NaCl, 1 mM EDTA, 1% Triton X-100, 0.1% sodium deoxycholate, 0.1% SDS, 1 mM phenylmethylsulfonyl fluoride), and resuspended in 1 ml of cold FA-SDS buffer. An equal volume of glass beads (0.5 mm in diameter) was added, and the cells were disrupted by vortexing for 30 min in a cold room. The lysate was diluted into 4 ml of FA-SDS buffer, and the glass beads were discarded. The cross-linked chromatin was then pelleted by centrifugation (17,000 × g for 35 min), washed for 60 min with FA-SDS buffer, resuspended in 1.6 ml of FA-SDS buffer for 15 min at 4°C, and sonicated three times for 30 s. each (Bioruptor, Diagenode), giving fragments with an average size of 250–300 bp. Finally, the sample was clarified by centrifugation at 14,000 × g for 30 min and diluted 4-fold in FA-SDS buffer, and aliquots of the resultant chromatin containing solution were stored at –80°C. Pol II and Myc^13^-tagged proteins were immunoprecipitated by incubating 100 μl of the chromatin containing solution for 180 min at 4°C with 2 μl of mouse anti-Pol II and anti-Myc antibodies, respectively (SCBT CTD4H8 or SC-40, respectively) prebound to 10 μl of Dynabeads Pan Mouse IgG (Dynal) according to the manufacturer's instructions. Immune complexes were washed six times in FA-SDS buffer and recovered by treating with 50 μl of Pronase Buffer (25 mM Tris, pH 7.5, 5 mM EDTA, 0.5% SDS) at 65°C with agitation. Input (IN) and immunoprecipitated (IP) fractions were then subjected to Pronase treatment (0.5 mg/ml; Roche Applied Science) for 60 min at 37°C, and formaldehyde cross-links were reversed by incubating the eluates overnight at 65°C. Finally, the samples were treated with RNase (50 μg/ml) for 60 min at 37°C. DNA from the IP fractions was purified using the High Pure PCR Product Purification Kit (Roche Applied Science) and eluted in 50 μl of 20 mM Tris buffer, pH 8. IN fractions were boiled 10 min and diluted 500-fold with no further purification prior to quantitative PCR analysis.

### Quantitative RT-PCR

Quantitative RT-PCR was performed as described previously [40] using primers listed in S11 Table. Total RNA was extracted from 4-ml cultures and cDNA was generated from 100 to 500 ng of total RNA using a RevertAid H Minus first-strand cDNA synthesis kit with oligo(dT)_18_ primers from Fermentas using the manufacturer's recommended protocol. cDNAs were subsequently quantified by RT-PCR using the Maxima SYBR green qPCR master mix from Fermentas.

### Co-immunoprecipitation

Cultures (100 ml) were harvested, washed once in 50 mM Tris, pH 8, and resuspended in 1ml of buffer (50 mM Tris, pH 8, 150 mM NaCl, 5 mM EDTA, 0.05% NP-40, 1 mM phenylmethylsulfonyl fluoride, and complete protease inhibitor cocktail tablets [Roche]). Lysis was performed by shaking with 425–600 μm acid-washed glass beads (Sigma) on an IKA Vibrax VXR orbital shaker at maximum speed for 30 min at 4°C. Cell debris and glass beads were removed by centrifugation. Immunoprecipitation was performed by incubating 200 μl of total cell extracts with 20 μl of Dynabeads PAN mouse immunoglobulin G (Invitrogen) that were preincubated with anti-HA (SCBT, SC-7392), anti-CTD (SCBT, CTD4H8), anti-Ser2P (BioLegend, H5) or anti-Ser5P (BioLegend, H14) antibodies and 20 μl of 1% phosphate-buffered saline-bovine serum albumin for 2 h under orbital shaking (800 rpm) at 30°C. Immune complexes were washed three times in lysis buffer, eluted by boiling in sodium dodecyl sulfate (SDS) sample buffer, and loaded on SDS-polyacrylamide gel for anti-Myc Western blotting.

### ChIP-Seq analysis and peak-calling

ChIP-Seq analysis was performed from two biological replicates of proline-grown 25T0b (no tag), FV078 (*DAL80-MYC*^*13*^) and FV034 (*GAT1-MYC*^*13*^) cells. Lysis and chromatin extraction was as described above. The average fragment length of sonicated fragment was 300–350 bp. For each condition, libraries were prepared from 10 ng of “input” or “IP” DNA using the TruSeq ChIP Sample Preparation Kit (Illumina). Single-read sequencing (50 nt) of the libraries was performed on a HiSeq 2500 sequencer.

Reads were uniquely mapped to the *S*. *cerevisiae* S288C reference genome using Bowtie2 v2.1.0 [89], with a tolerance of 1 mismatch in seed alignment. Tags densities were normalized on the total number of uniquely reads mapped.

Dal80- and Gat1-bound regions were identified through a peak-calling procedure using version 2.0.9 of MACS [90], with a minimum false discovery rate (FDR) of 0.001.

### Total RNA-Seq

For each strain and condition, total RNA was extracted from two biological replicates using standard hot phenol procedure, ethanol-precipitated, resuspended in nuclease-free H_2_O (Ambion) and quantified using a NanoDrop 2000c spectrophotometer. Ribosomal RNAs were depleted from 1 μg of total RNA using the RiboMinus Eukaryote v2 Kit (Life Technologies). After concentration using the Ribominus Concentration Module (Life Technologies), rRNA-depleted RNA was quantified using the Qubit RNA HS Assay kit (Life Technologies). In parallel, rRNA depletion efficiency and integrity of both total and rRNA-depleted RNA were checked by analysis in a RNA 6000 Pico chip, in a 2100 bioanalyzer (Agilent). Strand-specific total RNA-Seq libraries were prepared from 125 ng of rRNA-depleted RNA using the TruSeq Stranded Total RNA Sample Preparation Kit (Illumina), following manufacturer’s instructions. Paired-end sequencing (2 x 50 nt) of the libraries was performed on a HiSeq 2500 sequencer. Sequenced reads were mapped to the reference genome using version 2.0.6 of TopHat [91], as described [92]. Tags densities were normalized on the total number of reads uniquely mapped on ORFs. Differential expression analysis was performed using DESeq [93]. Differentially expressed genes were identified on the basis of a fold-change ≥2 and a *P*-value ≤0.01.

### Quantification and statistical analysis

Statistical details can be found in the corresponding figure legends. Error bars correspond to standard error. Statistical significance tests were carried out using the Student’s t test when indicated.

### Availability of data and materials

Sequence data can be accessed at the NCBI Gene Expression Omnibus using accession numbers GSE86307 and GSE86325. Genome browsers for visualization of processed ChIP-Seq and RNA-Seq data are accessible at http://vm-gb.curie.fr/dal80.

Further information and requests for resources and reagents should be directed to and will be fulfilled by the lead contact, Isabelle Georis (igeoris@ulb.ac.be). Bioinformatics and genome wide dataset requests could also be addressed to antonin.morillon@curie.fr for rapid processing.

## Supporting information

S1 Fig Related to Fig 1. **Genome-wide identification of Dal80-bound promoters**.
(A) Functionality of Dal80-Myc^13^. WT (25T0b), *dal80Δ* (FV080) and *DAL80-MYC*^*13*^ (FV078) cells were grown in glutamine- (Gln) or proline- (Pro) containing medium to mid-log phase. After total RNA isolation, levels of *DAL5* mRNA were quantified by qRT-PCR (primers Dal5_O9-O10_) and normalized on *SPT15* (alias *TBP1*) mRNA levels (primers SPT5_O1-O2_). Histograms represent the average of at least 2 independent experiments and the associated error bars correspond to the standard error.
(B) Box-plot of the distance between the annotated TSS and ORF start site (translation initiation codon, ATG) for protein-coding genes.
(C) Proportion of Dal80-bound and -unbound genes containing at least a GATA cluster in the promoter (-500 to -1 region, relative to the ATG codon of the downstream ORF). A GATA cluster is constituted by at least two GATA sites (GATAA, GATAAG or GATTAG), 15–35 bp apart.
(D) Orientation of GATA sites in the clusters defined above in Dal80-bound and -unbound promoters. The proportion of clusters containing GATA sites in head-to-head (H-H), head-to-tail (H-T), tail-to-head (T-H) and tail-to-tail (T-T) is shown for each class of promoters.
(E) Snapshot of ChIP-Seq signals along a GATA-less *locus* (*ALD6*). Densities (tag/nt) are shown for the untagged (black line) and *DAL80-MYC*^*13*^ (blue line) strains. Genes are represented as grey arrows. The region (70 bp) showing the maximum of Dal80-Myc^13^ binding is highlighted using the dashed box, and the corresponding sequence is shown below. The degenerated GATA sites (1 mismatch/motif) are highlighted in red, and stars indicate the residues that differ from the consensus. The snapshot was produced using the VING software [94].
(PPTX)

S2 Fig Related to Fig 2. **Dal80 recruitment to promoters correlates with nitrogen- and Dal80-sensitive gene expression**.
(A) Snapshot of RNA-Seq signals for the *DAL80* gene in WT-cells grown in glutamine- containing (Glu) or proline-containing (Pro) medium, and in *dal80Δ* cells grown in proline-containing medium. RNA-Seq signals are visualized as a heatmap. The upper and lower panels show the signals for the + and—strands, respectively. The color turns from yellow to dark blue as the signal increases (scale on the right). *DAL80* is highlighted using a dashed red box. The snapshot was produced using the VING software [94].
(B) Contingency table showing the number of Dal80-activated, -repressed and -insensitive genes among the (rev)NCR-sensitive and -insensitive genes. The results that were experimentally observed and those that are expected in case of independence are indicated in bold and in brackets, respectively. *P* < 0.00001 upon Chi-square test of independence.
(C) Contingency table showing the number of NCR-sensitive, revNCR-sensitive and unaffected genes among the Dal80-bound and unbound genes. The results that were experimentally observed and those that are expected in case of independence are indicated in bold and in brackets, respectively. *P* < 0.00001 upon Chi-square test of independence.
(D) Contingency table showing the number of Dal80-activated, Dal80-repressed and -insensitive genes among the Dal80-bound and unbound genes. The results that were experimentally observed and those that are expected in case of independence are indicated in bold and in brackets, respectively. *P* < 0.00001 upon Chi-square test of independence.
(PPTX)

S3 Fig Related to Fig 2. **Dal80 recruitment to promoters correlates with nitrogen- and Dal80-sensitive gene expression**.
(A) Snapshot of RNA-Seq signals for the *MEP2* gene in WT-cells grown in glutamine- containing (Glu) or proline-containing (Pro) medium, and in *dal80Δ* cells grown in proline-containing medium. RNA-Seq signals are visualized as described in S2A Fig. *MEP2* is highlighted using a dashed red box. The snapshot was produced using the VING software [94].
(B) Pol II occupancy at the *MEP2 locus*. WT (23344c) and *dal80Δ* (FV080) cells were grown in glutamine- (Gln) and/or proline-containing (Pro) medium. Anti-Pol II (CTD4H8) ChIP-qPCR analysis was performed using MEP2_P5-P6_, MEP2_P9-P10_, MEP2_O11-O12_ and MEP2_O9-O10_ primers. Histograms represent the averages of at least 2 independent experiments and the associated error bars correspond to the standard error.
(PPTX)

S4 Fig Related to Fig 4. **Dal80 spreading across gene bodies correlates with high expression levels**.
(A) Contingency table showing the number of NCR-sensitive, revNCR-sensitive and unaffected genes among the “P”, “P&O” and unbound genes. The results that were experimentally observed and those that are expected in case of independence are indicated in bold and in brackets, respectively. *P* < 0.00001 upon Chi-square test of independence.
(B) Contingency table showing the number of Dal80-activated, -repressed and–insensitive genes among the “P”, “P&O” and unbound genes. The results that were experimentally observed and those that are expected in case of independence are indicated in bold and in brackets, respectively. *P* < 0.00001 upon Chi-square test of independence.
(C) Density-plot of RNA-Seq signal (tag/nt, log2 scale) in WT cells grown in proline-containing medium, for genes of the “unbound” (blue, n = 4484), “P” (red, n = 1125) and P&O” (black, n = 144) classes. Y-axis: proportion of genes for each class. The highlighted areas correspond to the 75 (2%) and 170 (15%) genes of the “unbound” and “P” classes, respectively, showing a signal higher than the median of the “P&O” class. A box-plot representation of the same RNA-Seq signals is shown on the top of the density-plot.
(D) Same as above, highlighting the 949 (21%) and 632 (56%) genes of the “unbound” and “P” classes, respectively, showing a signal higher than the first quartile value for the “P&O” class.
(E) Venn diagram showing the number of genes of the “P” class (Dal80 binding restricted to the promoter) vs the *loci* previously defined as hyper-ChIPable [65].
(F) Same as above for the “P&O” class.
(G) Venn diagram showing the number of promoters bound by Dal80 and Gat1. Within each group, the number of *loci* previously defined as hyper-ChIPable [65] is indicated in red.
(H) Venn diagram showing the number of genes showing promoter and gene body binding (“P&O”) for Dal80 and Gat1. Within each group, the number of *loci* previously defined as hyper-ChIPable [65] is indicated in red.
(PPTX)

S5 Fig Related to Fig 6. **Dal80 occupancy across gene bodies requires active transcription and correlates with Pol II occupancy**.
(A) Snapshot of ChIP-Seq signals along the *UGA4 locus*. Densities (tag/nt) are shown for the untagged (25T0b; black line) and *DAL80-MYC*^*13*^ (FV078; blue line) strains. Genes and tRNA are represented as grey and black arrows, respectively. The snapshot was produced using the VING software [94].
(B) *UGA4* expression in a *DAL80ΔLZ* mutant strain. WT (25T0b), *DAL80-MYC*^*13*^ (FV078), *dal80Δ* (FV080) and *DAL80ΔLZ-MYC*^*13*^ (FV136) cells were grown in proline-containing medium. Total RNA was isolated and *UGA4* mRNA levels were quantified by qRT-PCR using UGA4_O1-O2_ primers as in S1A Fig.
(C) Occupancy of the *UGA4 locus* by Pol II. WT (25T0b), *DAL80-MYC*^*13*^ (FV078), *dal80Δ* (FV080) and *DAL80ΔLZ-MYC*^*13*^ (FV136) cells were grown in proline-containing medium. ChIP-qPCR analysis was performed as described in S3B Fig, using UGA4_O1-O2_ primers.
(PPTX)

S6 Fig Related to Fig 7. **Dal80 occupancy within gene bodies requires NCR promoter binding and correlates with Pol II occupancy**. *URA3* expression was determined in untagged wild type (25T0b), P_*MEP2*_-*URA3* (FV806), *DAL80-MYC*^*13*^ wild type (FV078) and P_*MEP2*_-*URA3* (FV808) cells grown in glutamine- or proline-containing medium. RT-qPCR analysis was performed as described in S1A Fig, using the URA3_O1-O2_ primers.
(PPTX)

S7 Fig Related to Fig 8. **Dal80 binding across *MEP2* requires active transcription**.
(A) Effect of Pol II elongation defects on *MEP2* expression. WT (FV673) or *rpb1-1* (FV675) *DAL80-MYC*^*13*^ cells were grown in glutamine- (Gln) or proline- (Pro) containing medium at 29°C to mid-log phase, then shifted at 37°C for one hour. Total RNA was isolated and *SPT15*-normalized *MEP2* mRNA levels were quantified by qRT-PCR using MEP2_O9-O10_ primers as in S1A Fig.
(B) Pol II occupancy at the *MEP2 locus* in *rpb1-1* cells. Wild type (FV673) or *rpb1-1* (FV675) *DAL80-MYC*^*13*^ cells were grown to mid-log phase at 29°C in the presence of glutamine (Gln) or proline (Pro) as unique nitrogen sources, and shifted at 37°C for one hour. ChIP analysis was conducted as described in S3B Fig, using MEP2_O9-O10_ primers.
(PPTX)

S1 Table Genome-wide identification of Dal80-bound promoters. List of 1269 gene promoters bound by Dal80.
(XLSX)

S2 Table Genome-wide identification of Dal80-bound promoters. GO term analysis of 1269 Dal80-bound promoters. Overlap of genes identified in our screens and in previous genome-wide expression screens.
(XLSX)

S3 Table Dal80 recruitment to promoters correlates with Dal80-sensivitiy. Lists of genes identified in ChIP-Seq (Column A) and RNA-Seq (Column C) analyses, or identified in previous screens (Column G + references in columns H-P), and their overlap (Columns B, D-F).
(XLSX)

S4 Table Dal80 recruitment to promoters correlates with nitrogen-sensitivity. List of the 754 genes upregulated (NCR-sensitive; column A) and 928 downregulated (revNCR-sensitive; column F) in a wild type (WT) grown in glutamine (G) or proline (P)-containing medium. Gene counts (Columns B-C and G-H), fold change (Columns D and I) and p values (Columns E and J) are indicated. Genes identified in previous screens (Column M + references in columns N-V) are indicated, and their overlap with our data (Columns K-L)
(XLSX)

S5 Table Dal80 recruitment to promoters correlates with Dal80-sensivitiy. List of 546 Dal80-regulated genes on proline: 314 genes are activated (Column A) and 232 genes are repressed (Column B). The list of genes having Gcn4 binding sites in their promoter is indicated (Column C), as well as its intersection with the activated gene list (Column D).
(XLSX)

S6 Table Dal80 recruitment to promoters correlates with Dal80-sensivitiy. Gene ontology term analysis of the 232 genes repressed by Dal80 on proline.
(XLSX)

S7 Table Dal80 recruitment to promoters correlates with Dal80-sensivitiy. Gene ontology term analysis of the 314 genes activated by Dal80 on proline.
(XLSX)

S8 Table Dal80 occupancy across the intragenic region of a subset of genes. Inventory of gene promoters and ORFs bound by Dal80 (Columns A-E) and their intersection with susceptibility to Dal80 regulation (Columns G-K).
(XLSX)

S9 Table Gat1 and Dal80 occupancy across the intragenic region of a subset of genes. List of gene promoters and P&O bound by Dal80-Myc^13^ (Columns A-B) or Gat1-Myc^13^ (Columns C-D) and their intersection (Columns E-F). The list of hyper-ChIPable genes is also provided (Column G) and intersection with Dal80- or Gat1-bound gene lists (Columns H-K).
(XLSX)

S10 Table Strains used in this study. (XLSX)

S11 Table Primers used in this study. (XLSX)

S1 Data Numerical data that underlie graphs and associated statistics. (XLSX)
