# Supplementary material for: Transcription-dependent spreading of the Dal80 yeast GATA factor across the body of highly expressed genes
Source: PLoS Genet. 2019 Feb 28;15(2):e1007999. doi: 10.1371/journal.pgen.1007999 (PMC6413948; doi:10.1371/journal.pgen.1007999)
Supplement: S1 Fig — Genome-wide identification of Dal80-bound promoters. (A) Functionality of Dal80-Myc13. WT (25T0b), dal80Δ (FV080) and DAL80-MYC13 (FV078) cells were grown in glutamine- (Gln) or proline- (Pro) containing medium to mid-log phase. After total RNA isolation, levels of DAL5 mRNA were quantified by qRT-PCR (primers Dal5O9-O10) and normalized on SPT15 (alias TBP1) mRNA levels (primers SPT5O1-O2). Histograms represent the average of at least 2 independent experiments and the associated error bars correspond to the standard error. (B) Box-plot of the distance between the annotated TSS and ORF start site (translation initiation codon, ATG) for protein-coding genes. (C) Proportion of Dal80-bound and -unbound genes containing at least a GATA cluster in the promoter (-500 to -1 region, relative to the ATG codon of the downstream ORF). A GATA cluster is constituted by at least two GATA sites (GATAA, GATAAG or GATTAG), 15–35 bp apart. (D) Orientation of GATA sites in the clusters defined above in Dal80-bound and -unbound promoters. The proportion of clusters containing GATA sites in head-to-head (H-H), head-to-tail (H-T), tail-to-head (T-H) and tail-to-tail (T-T) is shown for each class of promoters. (E) Snapshot of ChIP-Seq signals along a GATA-less locus (ALD6). Densities (tag/nt) are shown for the untagged (black line) and DAL80-MYC13 (blue line) strains. Genes are represented as grey arrows. The region (70 bp) showing the maximum of Dal80-Myc13 binding is highlighted using the dashed box, and the corresponding sequence is shown below. The degenerated GATA sites (1 mismatch/motif) are highlighted in red, and stars indicate the residues that differ from the consensus. The snapshot was produced using the VING software [94]. (PPTX) [file pgen.1007999.s001.pptx]

## Slide 1
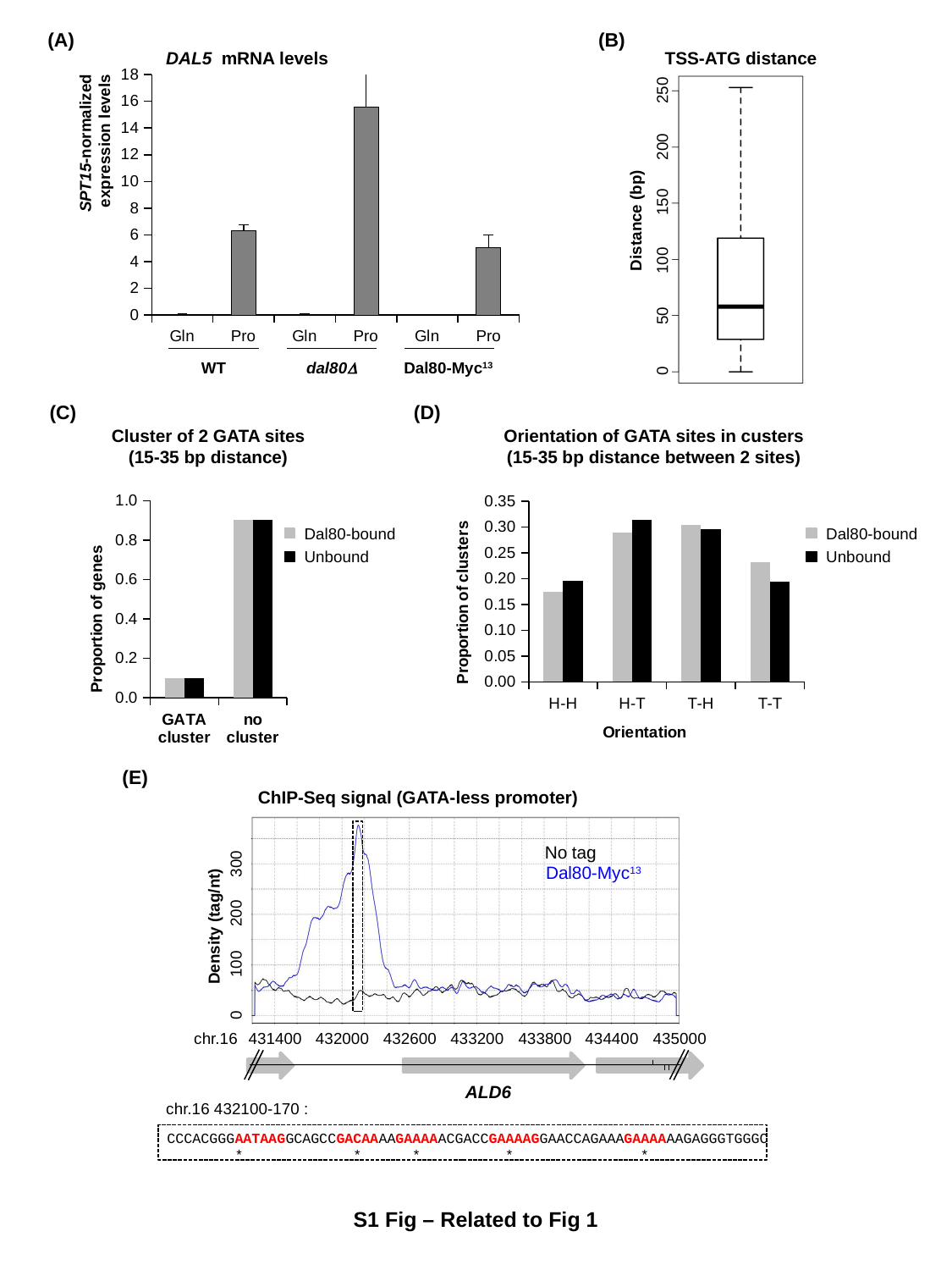

(A)
(B)
TSS-ATG distance
250
200
150
Distance (bp)
100
50
0
DAL5 mRNA levels
### Chart
| Category | |
|---|---|
| Gln | 0.0467734305067684 |
| Pro | 6.296709165641257 |
| Gln | 0.046128966034811314 |
| Pro | 15.578132301289692 |
| Gln | 0.03879722048936027 |
| Pro | 5.022243886447248 |SPT15-normalized expression levels
WT
dal80D
Dal80-Myc13
(C)
(D)
Cluster of 2 GATA sites
(15-35 bp distance)
Orientation of GATA sites in custers
(15-35 bp distance between 2 sites)
### Chart
| Category | Dal80-bound | Unbound |
|---|---|---|
| GATA cluster | 0.09692671394799054 | 0.09847648487524842 |
| no cluster | 0.903073286052009 | 0.9015235151247515 |
### Chart
| Category | Dal80-bound | Unbound |
|---|---|---|
| H-H | 0.17391304347826148 | 0.19635627530364375 |
| H-T | 0.289855072463768 | 0.313765182186235 |
| T-H | 0.3043478260869563 | 0.2955465587044535 |
| T-T | 0.231884057971014 | 0.1943319838056682 |Dal80-bound
Unbound
Dal80-bound
Unbound
(E)
ChIP-Seq signal (GATA-less promoter)
No tag
300
Dal80-Myc13
200
Density (tag/nt)
100
0
chr.16
431400
432000
432600
433200
433800
434400
435000
ALD6
chr.16 432100-170 :
CCCACGGGAATAAGGCAGCCGACAAAAGAAAAACGACCGAAAAGGAACCAGAAAGAAAAAAGAGGGTGGGC
 * * * * *
S1 Fig – Related to Fig 1
