# Supplementary material for: Transcription-dependent spreading of the Dal80 yeast GATA factor across the body of highly expressed genes
Source: PLoS Genet. 2019 Feb 28;15(2):e1007999. doi: 10.1371/journal.pgen.1007999 (PMC6413948; doi:10.1371/journal.pgen.1007999)
Supplement: S2 Fig — Dal80 recruitment to promoters correlates with nitrogen- and Dal80-sensitive gene expression. (A) Snapshot of RNA-Seq signals for the DAL80 gene in WT-cells grown in glutamine- containing (Glu) or proline-containing (Pro) medium, and in dal80Δ cells grown in proline-containing medium. RNA-Seq signals are visualized as a heatmap. The upper and lower panels show the signals for the + and—strands, respectively. The color turns from yellow to dark blue as the signal increases (scale on the right). DAL80 is highlighted using a dashed red box. The snapshot was produced using the VING software [94]. (B) Contingency table showing the number of Dal80-activated, -repressed and -insensitive genes among the (rev)NCR-sensitive and -insensitive genes. The results that were experimentally observed and those that are expected in case of independence are indicated in bold and in brackets, respectively. P < 0.00001 upon Chi-square test of independence. (C) Contingency table showing the number of NCR-sensitive, revNCR-sensitive and unaffected genes among the Dal80-bound and unbound genes. The results that were experimentally observed and those that are expected in case of independence are indicated in bold and in brackets, respectively. P < 0.00001 upon Chi-square test of independence. (D) Contingency table showing the number of Dal80-activated, Dal80-repressed and -insensitive genes among the Dal80-bound and unbound genes. The results that were experimentally observed and those that are expected in case of independence are indicated in bold and in brackets, respectively. P < 0.00001 upon Chi-square test of independence. (PPTX) [file pgen.1007999.s002.pptx]

## Slide 1
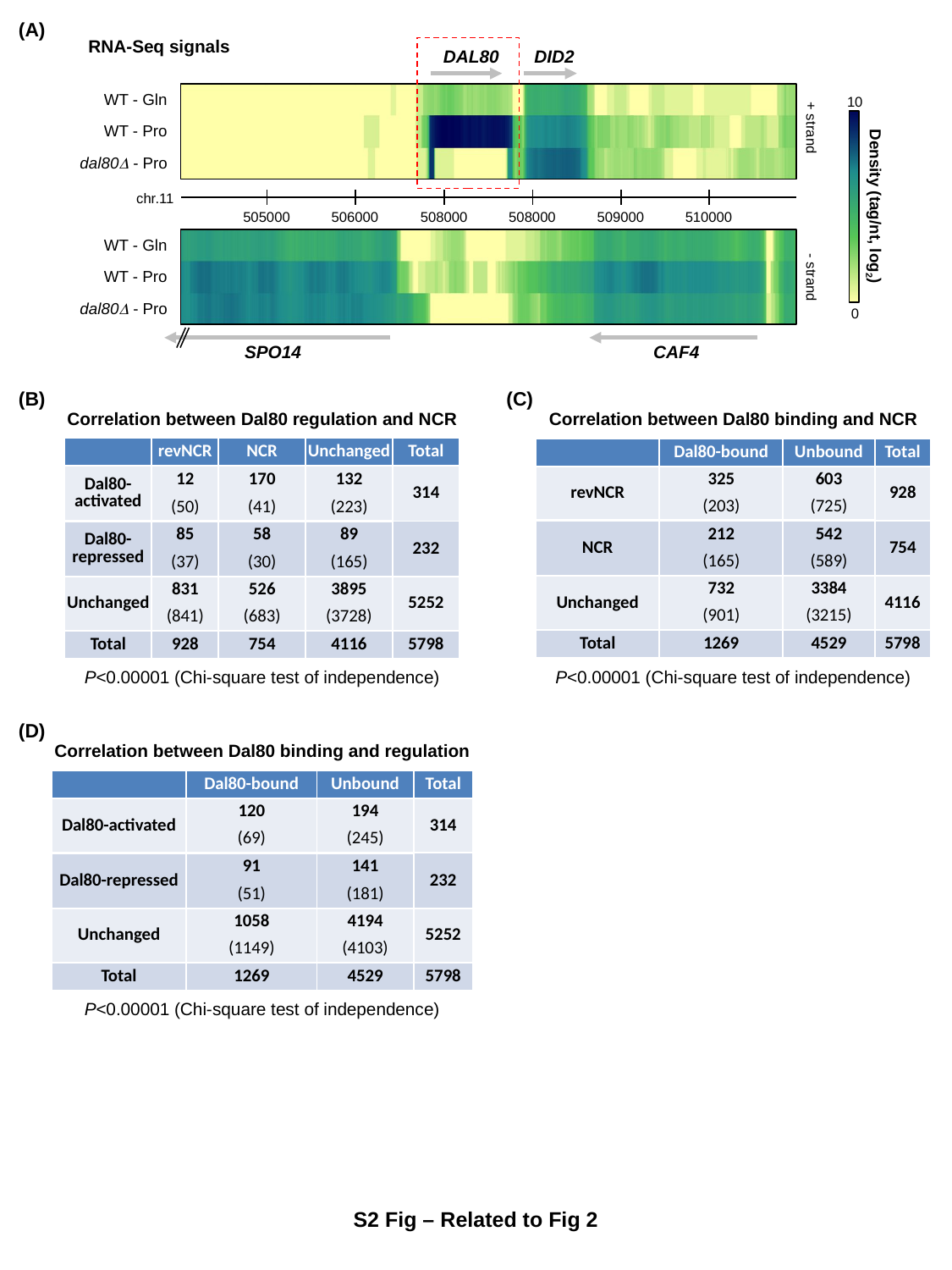

(A)
RNA-Seq signals
DAL80
DID2
WT - Gln
+ strand
WT - Pro
dal80D - Pro
chr.11
505000
506000
508000
508000
509000
510000
WT - Gln
WT - Pro
- strand
dal80D - Pro
CAF4
SPO14
0
10
Density (tag/nt, log2)
(B)
(C)
Correlation between Dal80 regulation and NCR
Correlation between Dal80 binding and NCR
| | revNCR | NCR | Unchanged | Total |
| --- | --- | --- | --- | --- |
| Dal80-activated | 12 | 170 | 132 | 314 |
| | (50) | (41) | (223) | |
| Dal80-repressed | 85 | 58 | 89 | 232 |
| | (37) | (30) | (165) | |
| Unchanged | 831 | 526 | 3895 | 5252 |
| | (841) | (683) | (3728) | |
| Total | 928 | 754 | 4116 | 5798 |
| | Dal80-bound | Unbound | Total |
| --- | --- | --- | --- |
| revNCR | 325 | 603 | 928 |
| | (203) | (725) | |
| NCR | 212 | 542 | 754 |
| | (165) | (589) | |
| Unchanged | 732 | 3384 | 4116 |
| | (901) | (3215) | |
| Total | 1269 | 4529 | 5798 |
P<0.00001 (Chi-square test of independence)
P<0.00001 (Chi-square test of independence)
(D)
Correlation between Dal80 binding and regulation
| | Dal80-bound | Unbound | Total |
| --- | --- | --- | --- |
| Dal80-activated | 120 | 194 | 314 |
| | (69) | (245) | |
| Dal80-repressed | 91 | 141 | 232 |
| | (51) | (181) | |
| Unchanged | 1058 | 4194 | 5252 |
| | (1149) | (4103) | |
| Total | 1269 | 4529 | 5798 |
P<0.00001 (Chi-square test of independence)
S2 Fig – Related to Fig 2
