# Supplementary material for: Transcription-dependent spreading of the Dal80 yeast GATA factor across the body of highly expressed genes
Source: PLoS Genet. 2019 Feb 28;15(2):e1007999. doi: 10.1371/journal.pgen.1007999 (PMC6413948; doi:10.1371/journal.pgen.1007999)
Supplement: S3 Fig — Dal80 recruitment to promoters correlates with nitrogen- and Dal80-sensitive gene expression. (A) Snapshot of RNA-Seq signals for the MEP2 gene in WT-cells grown in glutamine- containing (Glu) or proline-containing (Pro) medium, and in dal80Δ cells grown in proline-containing medium. RNA-Seq signals are visualized as described in S2A Fig. MEP2 is highlighted using a dashed red box. The snapshot was produced using the VING software [94]. (B) Pol II occupancy at the MEP2 locus. WT (23344c) and dal80Δ (FV080) cells were grown in glutamine- (Gln) and/or proline-containing (Pro) medium. Anti-Pol II (CTD4H8) ChIP-qPCR analysis was performed using MEP2P5-P6, MEP2P9-P10, MEP2O11-O12 and MEP2O9-O10 primers. Histograms represent the averages of at least 2 independent experiments and the associated error bars correspond to the standard error. (PPTX) [file pgen.1007999.s003.pptx]

## Slide 1
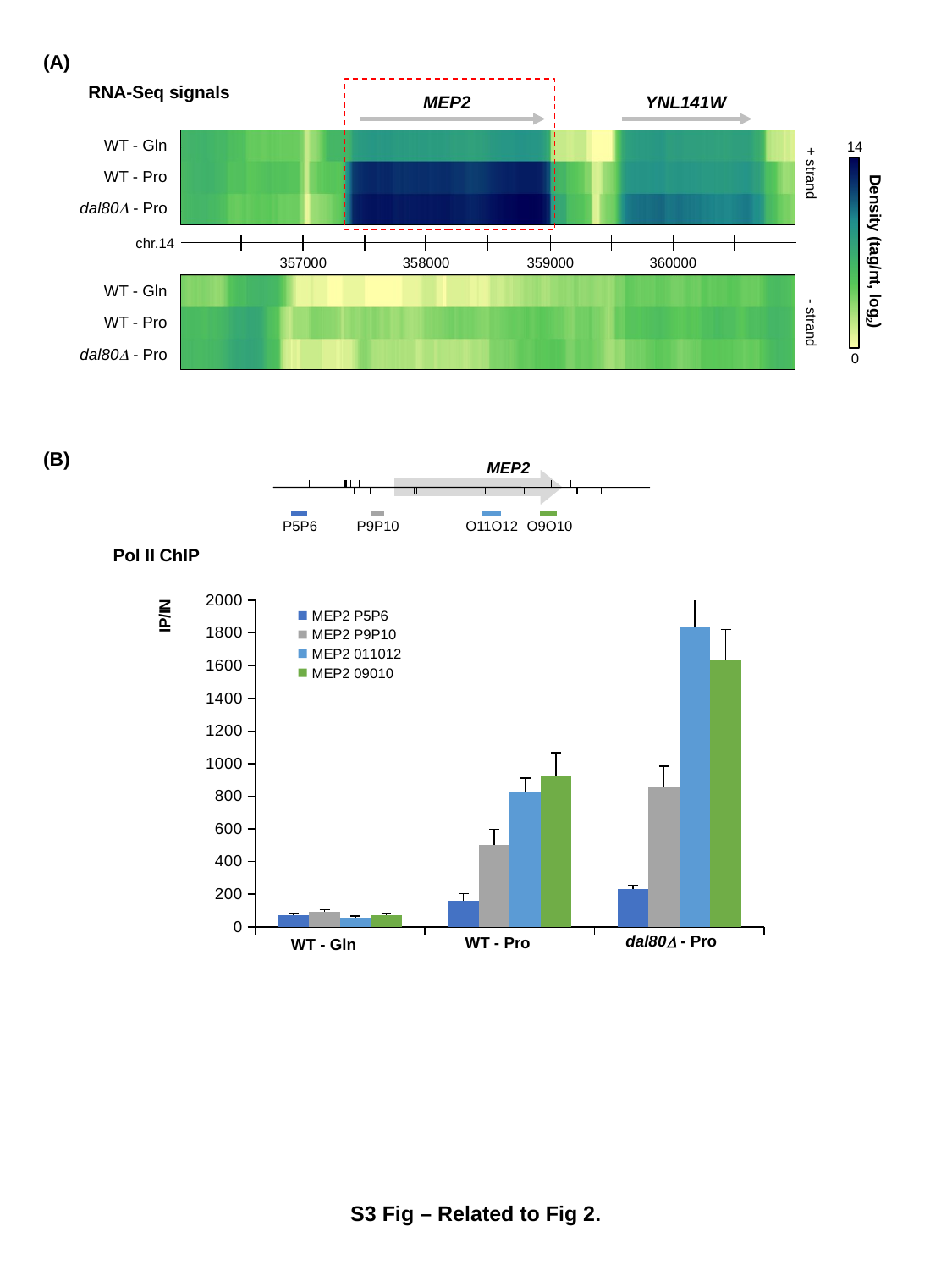

(A)
RNA-Seq signals
MEP2
YNL141W
WT - Gln
14
Density (tag/nt, log2)
0
+ strand
WT - Pro
dal80D - Pro
chr.14
357000
358000
359000
360000
WT - Gln
WT - Pro
- strand
dal80D - Pro
(B)
MEP2
P5P6
P9P10
O11O12
O9O10
Pol II ChIP
### Chart
| Category | MEP2 P5P6 | MEP2 P9P10 | MEP2 011012 | MEP2 09010 |
|---|---|---|---|---|
| WT Gln | 71.13315429588656 | 93.85194701523125 | 56.4939523877493 | 72.61585626758742 |
| WT Pro | 157.60437011875476 | 501.98338929409465 | 825.6717998928037 | 927.0987258830615 |
| dal80 Pro | 232.7512712929298 | 854.4751741437137 | 1831.5515715462966 | 1630.6803342362828 |dal80D - Pro
WT - Pro
WT - Gln
S3 Fig – Related to Fig 2.
