# Supplementary material for: Transcription-dependent spreading of the Dal80 yeast GATA factor across the body of highly expressed genes
Source: PLoS Genet. 2019 Feb 28;15(2):e1007999. doi: 10.1371/journal.pgen.1007999 (PMC6413948; doi:10.1371/journal.pgen.1007999)
Supplement: S4 Fig — Dal80 spreading across gene bodies correlates with high expression levels. (A) Contingency table showing the number of NCR-sensitive, revNCR-sensitive and unaffected genes among the “P”, “P&O” and unbound genes. The results that were experimentally observed and those that are expected in case of independence are indicated in bold and in brackets, respectively. P < 0.00001 upon Chi-square test of independence. (B) Contingency table showing the number of Dal80-activated, -repressed and–insensitive genes among the “P”, “P&O” and unbound genes. The results that were experimentally observed and those that are expected in case of independence are indicated in bold and in brackets, respectively. P < 0.00001 upon Chi-square test of independence. (C) Density-plot of RNA-Seq signal (tag/nt, log2 scale) in WT cells grown in proline-containing medium, for genes of the “unbound” (blue, n = 4484), “P” (red, n = 1125) and P&O” (black, n = 144) classes. Y-axis: proportion of genes for each class. The highlighted areas correspond to the 75 (2%) and 170 (15%) genes of the “unbound” and “P” classes, respectively, showing a signal higher than the median of the “P&O” class. A box-plot representation of the same RNA-Seq signals is shown on the top of the density-plot. (D) Same as above, highlighting the 949 (21%) and 632 (56%) genes of the “unbound” and “P” classes, respectively, showing a signal higher than the first quartile value for the “P&O” class. (E) Venn diagram showing the number of genes of the “P” class (Dal80 binding restricted to the promoter) vs the loci previously defined as hyper-ChIPable [65]. (F) Same as above for the “P&O” class. (G) Venn diagram showing the number of promoters bound by Dal80 and Gat1. Within each group, the number of loci previously defined as hyper-ChIPable [65] is indicated in red. (H) Venn diagram showing the number of genes showing promoter and gene body binding (“P&O”) for Dal80 and Gat1. Within each group, the number of loci previously defined a [file pgen.1007999.s004.pptx]

## Slide 1
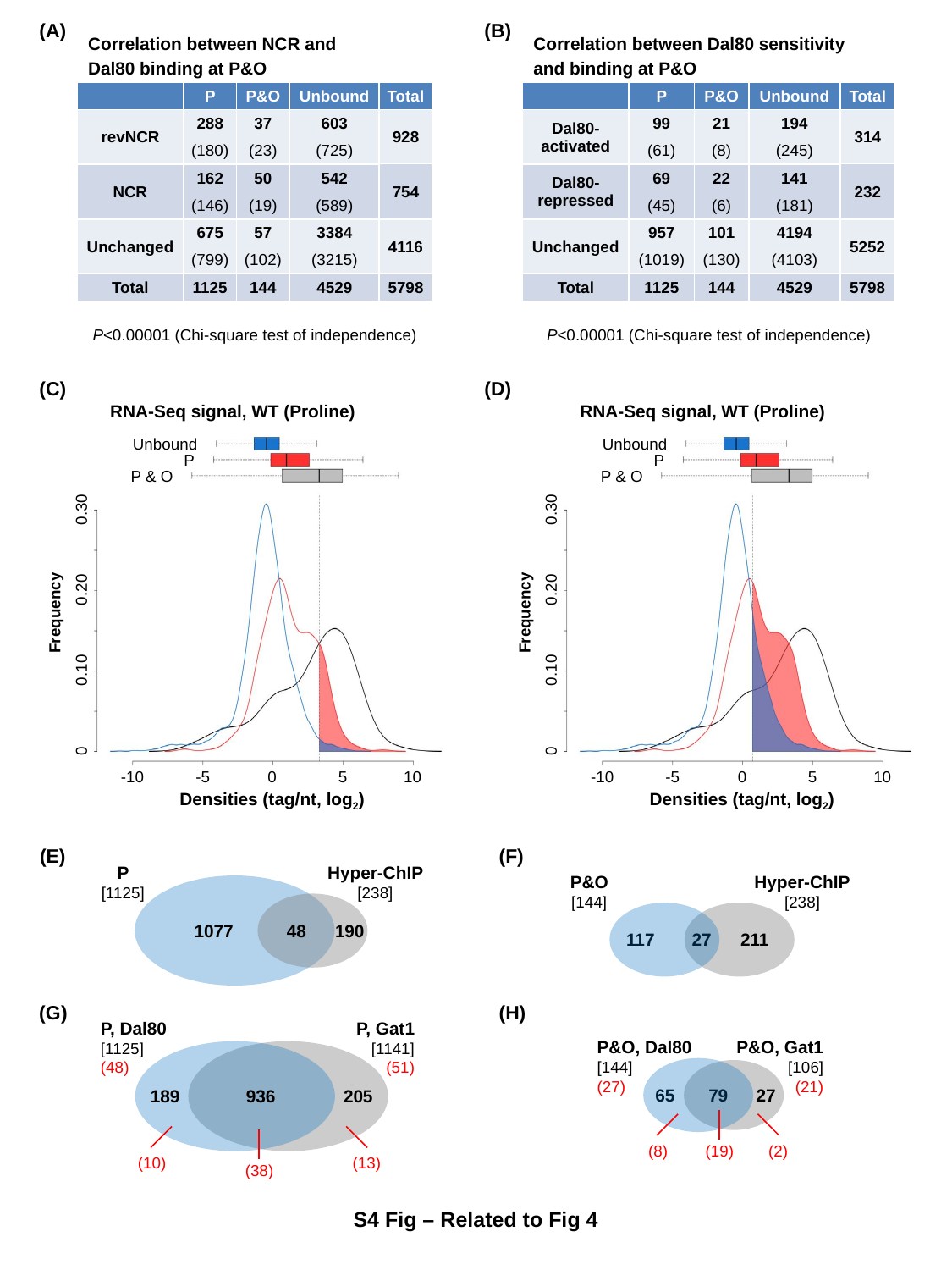

(A)
(B)
Correlation between NCR and
Dal80 binding at P&O
Correlation between Dal80 sensitivity
and binding at P&O
| | P | P&O | Unbound | Total |
| --- | --- | --- | --- | --- |
| revNCR | 288 | 37 | 603 | 928 |
| | (180) | (23) | (725) | |
| NCR | 162 | 50 | 542 | 754 |
| | (146) | (19) | (589) | |
| Unchanged | 675 | 57 | 3384 | 4116 |
| | (799) | (102) | (3215) | |
| Total | 1125 | 144 | 4529 | 5798 |
| | P | P&O | Unbound | Total |
| --- | --- | --- | --- | --- |
| Dal80-activated | 99 | 21 | 194 | 314 |
| | (61) | (8) | (245) | |
| Dal80-repressed | 69 | 22 | 141 | 232 |
| | (45) | (6) | (181) | |
| Unchanged | 957 | 101 | 4194 | 5252 |
| | (1019) | (130) | (4103) | |
| Total | 1125 | 144 | 4529 | 5798 |
P<0.00001 (Chi-square test of independence)
P<0.00001 (Chi-square test of independence)
(C)
(D)
RNA-Seq signal, WT (Proline)
Unbound
P
P & O
0.30
0.20
Frequency
0.10
0
-10
-5
0
5
10
Densities (tag/nt, log2)
RNA-Seq signal, WT (Proline)
Unbound
P
P & O
0.30
0.20
Frequency
0.10
0
-10
-5
0
5
10
Densities (tag/nt, log2)
(E)
(F)
P
[1125]
Hyper-ChIP
[238]
1077
48
190
P&O
[144]
Hyper-ChIP
[238]
117
27
211
(G)
(H)
P, Dal80
[1125]
(48)
P, Gat1
[1141]
(51)
189
936
205
(13)
(38)
(10)
P&O, Dal80
[144]
(27)
P&O, Gat1
[106]
(21)
65
79
27
(19)
(2)
(8)
S4 Fig – Related to Fig 4
