# Supplementary material for: Transcription-dependent spreading of the Dal80 yeast GATA factor across the body of highly expressed genes
Source: PLoS Genet. 2019 Feb 28;15(2):e1007999. doi: 10.1371/journal.pgen.1007999 (PMC6413948; doi:10.1371/journal.pgen.1007999)
Supplement: S5 Fig — Dal80 occupancy across gene bodies requires active transcription and correlates with Pol II occupancy. (A) Snapshot of ChIP-Seq signals along the UGA4 locus. Densities (tag/nt) are shown for the untagged (25T0b; black line) and DAL80-MYC13 (FV078; blue line) strains. Genes and tRNA are represented as grey and black arrows, respectively. The snapshot was produced using the VING software [94]. (B) UGA4 expression in a DAL80ΔLZ mutant strain. WT (25T0b), DAL80-MYC13 (FV078), dal80Δ (FV080) and DAL80ΔLZ-MYC13 (FV136) cells were grown in proline-containing medium. Total RNA was isolated and UGA4 mRNA levels were quantified by qRT-PCR using UGA4O1-O2 primers as in S1A Fig. (C) Occupancy of the UGA4 locus by Pol II. WT (25T0b), DAL80-MYC13 (FV078), dal80Δ (FV080) and DAL80ΔLZ-MYC13 (FV136) cells were grown in proline-containing medium. ChIP-qPCR analysis was performed as described in S3B Fig, using UGA4O1-O2 primers. (PPTX) [file pgen.1007999.s005.pptx]

## Slide 1
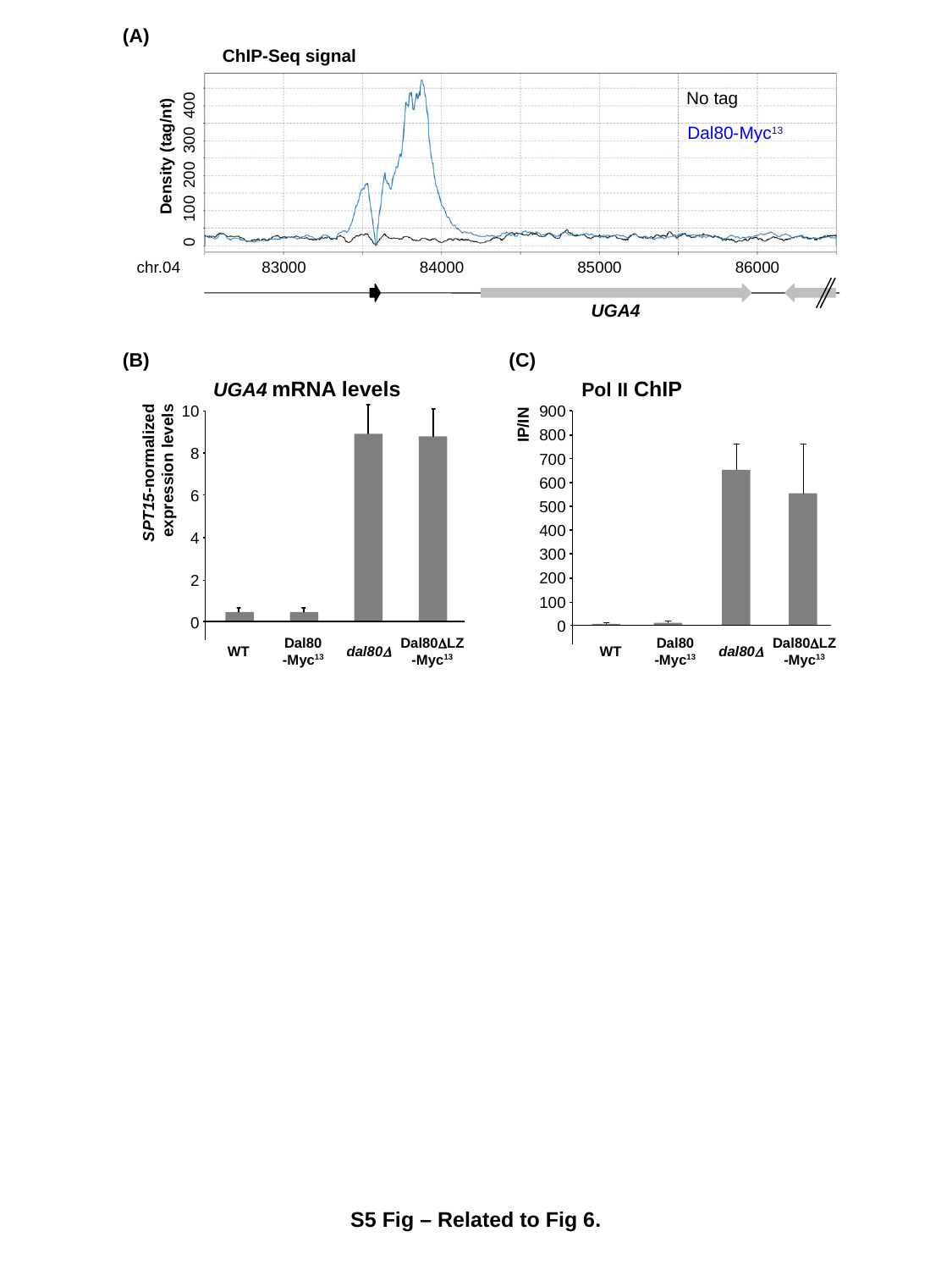

(A)
ChIP-Seq signal
No tag
400
Dal80-Myc13
300
Density (tag/nt)
200
100
0
chr.04
83000
84000
85000
86000
UGA4
(B)
(C)
UGA4 mRNA levels
Pol II ChIP
10
900
IP/IN
800
8
700
SPT15-normalized expression levels
600
6
500
400
4
300
200
2
100
0
0
Dal80
-Myc13
Dal80DLZ
-Myc13
Dal80
-Myc13
Dal80DLZ
-Myc13
WT
dal80D
WT
dal80D
S5 Fig – Related to Fig 6.
