# Supplementary material for: Transcription-dependent spreading of the Dal80 yeast GATA factor across the body of highly expressed genes
Source: PLoS Genet. 2019 Feb 28;15(2):e1007999. doi: 10.1371/journal.pgen.1007999 (PMC6413948; doi:10.1371/journal.pgen.1007999)
Supplement: S6 Fig — Dal80 occupancy within gene bodies requires NCR promoter binding and correlates with Pol II occupancy. URA3 expression was determined in untagged wild type (25T0b), PMEP2-URA3 (FV806), DAL80-MYC13 wild type (FV078) and PMEP2-URA3 (FV808) cells grown in glutamine- or proline-containing medium. RT-qPCR analysis was performed as described in S1A Fig, using the URA3O1-O2 primers. (PPTX) [file pgen.1007999.s006.pptx]

## Slide 1
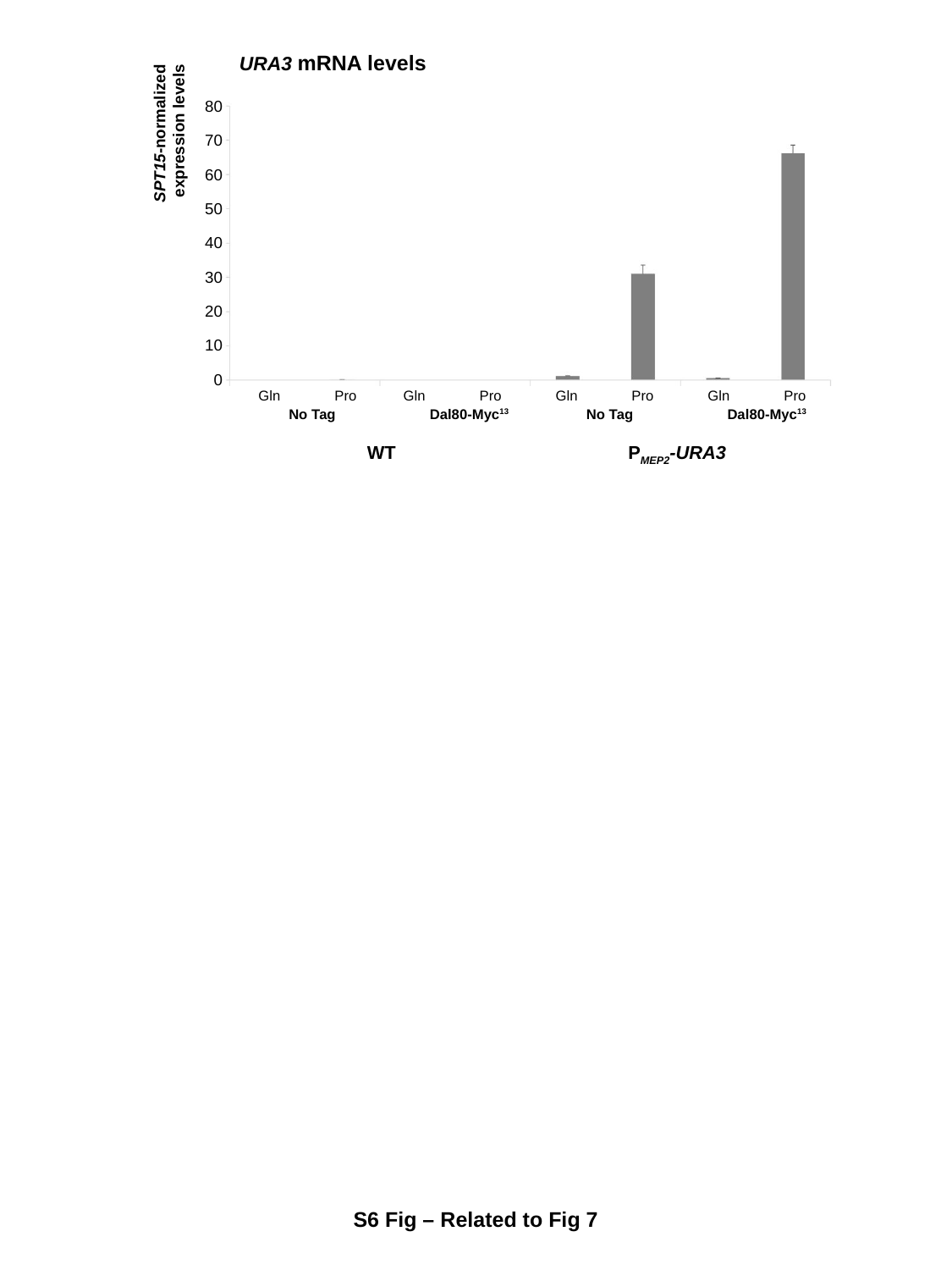

URA3 mRNA levels
80
SPT15-normalized expression levels
70
60
50
40
30
20
10
0
Gln
Pro
Gln
Pro
Gln
Pro
Gln
Pro
No Tag
Dal80-Myc13
No Tag
Dal80-Myc13
WT
PMEP2-URA3
S6 Fig – Related to Fig 7
