# Supplementary material for: Transcription-dependent spreading of the Dal80 yeast GATA factor across the body of highly expressed genes
Source: PLoS Genet. 2019 Feb 28;15(2):e1007999. doi: 10.1371/journal.pgen.1007999 (PMC6413948; doi:10.1371/journal.pgen.1007999)
Supplement: S7 Fig — Dal80 binding across MEP2 requires active transcription. (A) Effect of Pol II elongation defects on MEP2 expression. WT (FV673) or rpb1-1 (FV675) DAL80-MYC13 cells were grown in glutamine- (Gln) or proline- (Pro) containing medium at 29°C to mid-log phase, then shifted at 37°C for one hour. Total RNA was isolated and SPT15-normalized MEP2 mRNA levels were quantified by qRT-PCR using MEP2O9-O10 primers as in S1A Fig. (B) Pol II occupancy at the MEP2 locus in rpb1-1 cells. Wild type (FV673) or rpb1-1 (FV675) DAL80-MYC13 cells were grown to mid-log phase at 29°C in the presence of glutamine (Gln) or proline (Pro) as unique nitrogen sources, and shifted at 37°C for one hour. ChIP analysis was conducted as described in S3B Fig, using MEP2O9-O10 primers. (PPTX) [file pgen.1007999.s007.pptx]

## Slide 1
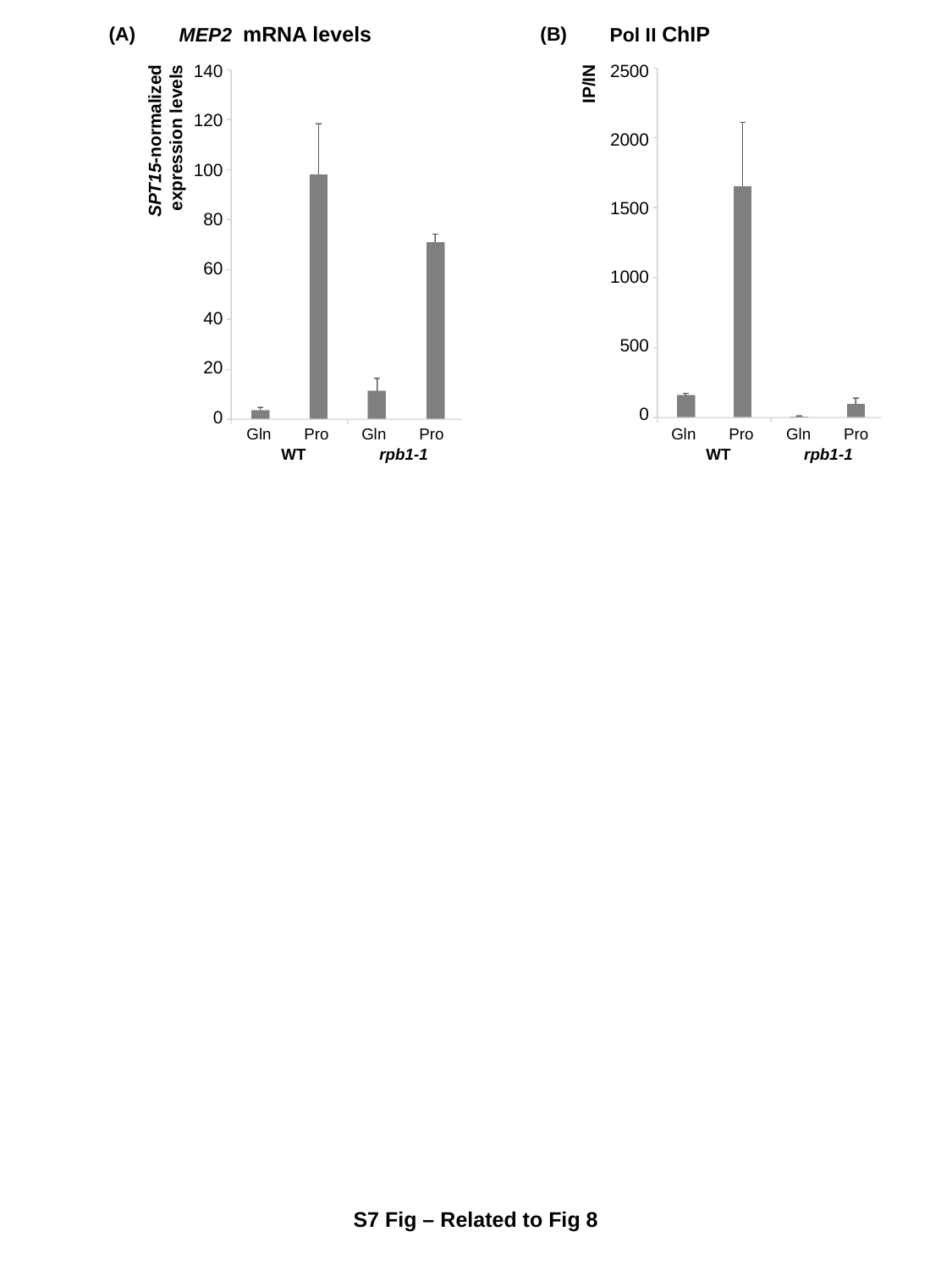

(A)
(B)
MEP2 mRNA levels
Pol II ChIP
140
2500
IP/IN
120
2000
SPT15-normalized expression levels
100
1500
80
60
1000
40
500
20
0
0
Gln
Pro
Gln
Pro
Gln
Pro
Gln
Pro
WT
rpb1-1
WT
rpb1-1
S7 Fig – Related to Fig 8
